# Potential Association Between Dietary Fibre and Humoral Response to the Seasonal Influenza Vaccine

**DOI:** 10.3389/fimmu.2021.765528

**Published:** 2021-11-17

**Authors:** Alissa Cait, Anna Mooney, Hazel Poyntz, Nick Shortt, Angela Jones, Aurélie Gestin, Katie Gell, Alix Grooby, David O’Sullivan, Jeffry S. Tang, Wayne Young, Darmiga Thayabaran, Jenny Sparks, Tess Ostapowicz, Audrey Tay, Sally D. Poppitt, Sarah Elliott, Georgia Wakefield, Amber Parry-Strong, Jacqui Ralston, Richard Beasley, Mark Weatherall, Irene Braithwaite, Elizabeth Forbes-Blom, Olivier Gasser

**Affiliations:** ^1^ Malaghan Institute of Medical Research, Wellington, New Zealand; ^2^ High-Value Nutrition National Science Challenge, Auckland, New Zealand; ^3^ Medical Research Institute of New Zealand, Wellington, New Zealand; ^4^ AgResearch, Palmerston North, New Zealand; ^5^ Human Nutrition Unit, Department of Medicine, School of Biological Sciences, University of Auckland, Auckland, New Zealand; ^6^ Food Savvy, Wellington, New Zealand; ^7^ Center for Endocrine, Diabetes and Obesity Research Capital & Coast District Health Board (CCDHB), Wellington, New Zealand; ^8^ Institute of Environmental Science and Research Limited (ESR), National Centre for Biosecurity and Infectious Disease (NCBID), Upper Hutt, New Zealand; ^9^ Wellington School of Medicine, University of Otago, Wellington, New Zealand

**Keywords:** fibre, microbiome, influenza, vaccine, short chain fatty acids (SCFA)

## Abstract

Influenza vaccination is an effective public health measure to reduce the risk of influenza illness, particularly when the vaccine is well matched to circulating strains. Notwithstanding, the efficacy of influenza vaccination varies greatly among vaccinees due to largely unknown immunological determinants, thereby dampening population-wide protection. Here, we report that dietary fibre may play a significant role in humoral vaccine responses. We found dietary fibre intake and the abundance of fibre-fermenting intestinal bacteria to be positively correlated with humoral influenza vaccine-specific immune responses in human vaccinees, albeit without reaching statistical significance. Importantly, this correlation was largely driven by first-time vaccinees; prior influenza vaccination negatively correlated with vaccine immunogenicity. In support of these observations, dietary fibre consumption significantly enhanced humoral influenza vaccine responses in mice, where the effect was mechanistically linked to short-chain fatty acids, the bacterial fermentation product of dietary fibre. Overall, these findings may bear significant importance for emerging infectious agents, such as COVID-19, and associated *de novo* vaccinations.

## Introduction

Vaccination is our best defence against severe illness and death caused by influenza viruses. However, immune responses to seasonal influenza vaccinations vary substantially in efficacy across the general population, leaving some individuals susceptible to infection each season, even after being vaccinated. The reason for this variation is mostly unknown.

The microbiota has been increasingly investigated in this context and found to contribute to the inter-individual variance in vaccine responses in mice and humans (1). There are now several lines of evidence that demonstrate a relationship between the gut microbiota and protective antibody responses during vaccination (2). Compelling animal studies have shown that a disrupted microbiome will impair vaccine-induced antibody responses (3–6). Despite this growing body of work, our understanding of whether the microbiome impacts these same immunological processes in humans is surprisingly under-studied. Only a limited number of clinical studies demonstrate correlations between microbiome features and impaired antibody responses (7–10), but mechanistic data is lacking.

Diet plays a substantial role in determining the composition of our microbiome and long-term dietary habits represent one of the most dominant environmental factors that influence gut microbiome composition (11). However, the potential links between dietary patterns of free-living, non-malnourished humans and vaccine responses have not yet been addressed or described. Of particular relevance in this context are dietary components that cannot be digested by the host, but are readily metabolized by resident microbes to support the symbiotic relationship between host and microbes (12, 13) – most importantly the microbial fibre fermentation products, short chain fatty acids (SCFAs) (14). The SCFAs with highest concentration in the healthy human gut are acetate (C2), propionate (C3), and butyrate (C4) (15). The relationship between dietary fibre, SCFAs, and antibody responses has been previously demonstrated to activate B cell metabolism and promote protective antibody responses during infection in a mouse model of *Citrobacter rodentium* infection (5). Whether dietary fibre similarly influences antibody responses in humans is currently unknown.

To understand the role of diet and the microbiome on antibody responses to the 2016 Trivalent Influenza Vaccine (TIV), Influvac^®^ (Mylan, Illinois, USA) we previously performed and reported on a 6-month observational study on New Zealand adults who received TIV, collecting data on gut microbiota composition, diet, and humoral vaccine response (16) (Australia and New Zealand Clinical Trials Registry number ACTRN1261500 1365550). Here, after conducting *ad hoc* analyses to assess the impact of dietary fibre intake on TIV-specific humoral immune responses, we identify a correlation between fibre consumption and vaccine responses in individuals who are naïve to previous influenza vaccination. The observed correlation did however not reach statistical significance. Furthermore, in a corresponding animal model of TIV vaccination, we demonstrate that fibre consumption or supplementation with SCFAs can mediate a comparable immunological outcome, indicating that fibre consumption and subsequent production of microbially derived SCFAs can improve TIV-specific humoral immune responses. Taken together, these findings reveal an unappreciated role for a diet rich in fibre in inducing a robust antibody response to seasonal influenza vaccine.

## Methods

### Clinical Study Design

122 healthy participants aged 18-64 received the 2016 trivalent influenza vaccine (TIV) as part of this study. Detailed study characteristics are described elsewhere (16). All participants who completed the study were included in our analysis, unless otherwise stated. Exclusion criteria included: *i*. a known severe reaction or allergy to any components of the influenza vaccine. *ii.* Any contraindications to vaccination per recommendations of the vaccine manufacturer. *iii.* A history of Guillain-Barre Syndrome within six weeks of receiving a previous influenza vaccine. *iv.* Any immune impairment that could confound immune testing. *v.* Already having received the 2016 seasonal influenza vaccine.

### Fecal Samples

Stool samples were collected by participants using the OMNIgene GUT collection kit (DNA Genotek, Ontario, Canada). Samples were collected by participants in the three-day period prior to both Day Zero and Day 28. For microbiota profiling, DNA was extracted from faecal samples using the Nucleospin Soil kit (Macherey-Nagel (Duren, Germany) following the manufacturer’s instructions. Briefly, 500 mg fecal sample was suspended in lysis buffer and mechanically disrupted using ceramic beads. Proteins and PCR inhibitors were then pelleted with the ceramic beads and the supernatant adjusted to DNA-binding conditions before being passed over Nucleospin soil column to bind the DNA. Residual substances were then removed by efficient washing. Finally, the DNA was eluted in 100 µl of RNAse-free water. DNA yield was assessed using the Quantus™ fluorometer (Promega, Madison, WA, USA) and DNA quality measured with the Nanodrop ND-1000 spectrophotometer (Thermofisher, Waltham, MA, USA). Amplification of the V1-V3 region of the 16S rRNA gene followed by 2 X 250 bp sequencing on the MiSeq platform was performed at NZ Genomics Ltd (NZGL) using the standard Illumina method (https://www.illumina.com/content/dam/illumina-support/documents/documentation/chemistry_documentation/16s/16s-metagenomic-library-prep-guide-15044223-b.pdf).

### Microbiome Data Processing

Sequence data was trimmed, quality filtered, and clustered at 97% identity into Operational Taxonomic Units (OTUs) following the mothur SOP pipeline (17). Representative OTU sequences were assigned taxonomic classification using a Bayesian classifier against the Ribosomal database project (RDP) database (18). This generated an average of 50380.98 reads per sample, median 49514 reads (**Supplementary Figure 1**), clustered into 148038 OTUs. Before filtering, alpha diversity (Shannon measure) was calculated and plotted using Phyloseq. Data was transformed to a relative abundance [x/sum(x)] and filtered in Phyloseq to remove OTUs unclassified beyond the kingdom bacteria and OTUs that did not occur 2 or more times in 10% or more of samples, reducing the number of OTUs to 471. Data was further analysed in R using custom scripts and plotted using ggplot2 (19).

### Microbiome qPCR

Bacterial DNA was extracted from faecal samples using QIAamp Fast DNA Stool Mini Kit according to the manufacturer’s instructions. DNA concentration and purity was measured on a ND-1000 Nanodrop spectrophotometer (Thermofisher Scientific). Samples were stored at -20°C until required.

Proportions of dominant bacteria in the faeces were determined by quantitative PCR as previously described (20). A qPCR reaction mix was prepared by combining per reaction; 10 μL SYBR green PCR master mix, 1 μL each of forward and reverse primers (**Supplementary Table 1**) and 6 μL of DPEC-treated H2O. To each well of an optical 96-well reaction plate with barcode, 18 μL of prepared qPCR reaction mix and 2 μL of the DNA sample (standardised to 1 ng/μL) were added. The plate was covered with an optical adhesive film and centrifuged for approximately 30 seconds at 500 x g. All samples were run in duplicate and each plate included an H2O sample as a negative control. qPCR was run on a QuantStudio 7 Flex PCR system (Thermofisher Scientific) using the standard instrument settings for fast TaqMan 96-well plate assay.

### Food Diaries

Self-reported dietary intake was assessed using a 7-day food diary. Participants recorded all meals and snacks consumed throughout 7 consecutive days, including beverages. Food diaries were analysed using the dietary software FoodWorks 7 (Xyris Software, Australia) at the Human Nutrition Unit of the University of Auckland, New Zealand. RCT design adherence was considered to be a reported daily energy intake of >1.2 x basal metabolic rate (BMR), using Schofield predicted BMR (21) based on gender, age, height and body weight of each participant at baseline.

### Vaccine History

Vaccine history was collected in the form of a questionnaire. Participants self-reported if they had received the seasonal influenza in 2015, 2014, 2013, 2012, 2011 *or earlier.*

### Hemagglutination Inhibition Assays

Influenza-specific antibody titres in serum were quantified using the HAI assay. Serum was separated from whole blood samples by centrifugation then stored at -70°C until use. HAI assays were performed for each influenza strain by the Institute of Environmental Science and Research Ltd, based at the National Centre for Biosecurity & Infectious Disease Upper Hutt, New Zealand. Briefly, serum samples were treated with receptor destroying enzyme and heat inactivated to remove nonspecific agglutinins. The samples were titrated out twofold, in duplicate, across a microtitre plate from 1:10 to 1:10240; then, four haemagglutinating units of the appropriate antigen were added. After a 30-min incubation step, 1% Guinea pig red blood cells (RBC) were added to all wells, including serum controls and test controls and were allowed to settle for an hour. Plates were read manually and titre endpoints determined as the last well where RBC agglutination was inhibited. Results were accepted if all controls (Serum and Antigen) gave expected results.

Participants were characterised into responder categories as follows: low responder if they had a <4 fold change for H1 and H3; medium responder if they had a ≥4 fold change for one strain and <4 fold change for the other strain; high responder if they had a ≥4 fold change for both H1 and H3.

### Neuraminidase Inhibition Assays

Influenza-specific antibody NAI titres in serum were quantified using the Enzyme-Linked Lectin assay. Serum was separated from whole blood samples by centrifugation then stored at -70°C until use. Enzyme linked lectin assays were performed for each influenza strain by the Institute of Environmental Science and Research Ltd, based at the National Centre for Biosecurity & Infectious Disease Upper Hutt, New Zealand. Briefly, serum samples were heat inactivated at 56°C for one hour then titrated out twofold, in duplicate, across a microtitre dilution plate, from 1:10 to 1:5120. Titrated samples were transferred to a fetuin-coated test plate, including controls, and a standard amount of antigen added. After overnight incubation, peroxidase conjugated peptide nucleic acid was added to the washed plates followed by steps in which colour was developed. Plates were read on a BioTek plate reader. The reduction or absence of colour indicated inhibition of NAI activity due to the presence of NA-specific antibodies.

### Mice

C57BL/6J mice were bred and maintained in the specific pathogen-free Biomedical Research Unit at the Malaghan Institute of Medical Research (Wellington, New Zealand). Mice were housed in autoclaved cages under specific pathogen free conditions. Mice were age and sex matched. Mice were used for experiments from 6-12 weeks of age. Both male and female mice were used. The Victoria University Animal Ethics Committee granted ethical approval for experimental animal procedures and all experiments were carried out in accordance with their guidelines. At experimental end points mice were humanely killed by CO_2_ inhalation followed by cardiac puncture.

### Mouse Diets

As indicated, breeding pairs and nursing dams were administered a zero-fibre diet (SF09-028, Specialty Feeds, Glen Forrest, Australia) or high fibre diet (7.5% Inulin, 7.5% Pectin SF15-086, Specialty Feeds). Pups born from respective breeding pairs were reared on this special diet for the duration of the experiment. All foods were autoclaved or irradiated prior to feeding for sterility.

Unless otherwise stated, mice were maintained on a meat-free rat and mouse standard diet. Other than the specified dietary alterations, paired diets in each experiment were designed to be otherwise nutritionally equivalent. Mice were administered foods *ad libitum* for the duration specified in each experiment.

### Broad-Spectrum Antibiotic Cocktail Administration to Mice

The broad-spectrum antibiotic cocktail (ABX) consisted of vancomycin at 0.5 mg/mL, neomycin at 1 mg/mL, ampicillin at 1 mg/mL and metronidazole at 1 mg/mL, dissolved in purified, acidified drinking water. This was administered to mice *ad libitum* for 14 days prior to TIV vaccination. Splenda artificial sweetener (4 mg/mL) and Hansell’s strawberry essence (2 μL/mL) were added to improve palatability of the solution. Splenda and Hansell’s strawberry essence were also added to the control water.

### Short-Chain Fatty Acid Administration to Mice

Solutions of single SCFAs were prepared by adding 200 mM of sodium acetate, sodium butyrate or sodium propionate (Sigma-Aldrich, St Louis, MO) to autoclaved drinking water, at a concentration of 200mM and administered to mice *ad libitum* for 21 days prior to vaccination and throughout the remainder of the experiment. A mixed SCFA solution was prepared by adding sodium acetate (70 mM), sodium butyrate (20 mM) and sodium propionate (30 mM), to autoclaved acidified water and was administered to mice *ad libitum* for 14 days prior to initial vaccination and throughout the remainder of the experiment.

### Trivalent Influenza Vaccine Administration to Mice

Influvac^®^ 2016 inactivated TIV vaccine was diluted 1:4 in sterile PBS and 200 μL was administered subcutaneously (SC) at the tail base between the dorsal and right lateral vein, with mice receiving 1/10 of the full human dose of 500 μL. Mice received this dose on day 0, 7, and 14. Mice were sacrificed on D21 to evaluate antibody responses.

### Serum TIV Enzyme Linked Immunosorbent Assays

Levels of TIV-specific IgG antibody in mouse serum were determined by ELISA. Briefly, each well of a 96 well Nunc Maxisorb plates was coated with 100 μL of Influvac^®^ diluted to 25 μL/mL in Phosphate Buffered Saline (PBS) and incubated overnight at 4°C. Non-specific protein binding was blocked with the addition of 150 μL of 10% fetal bovine serum in PBS and incubated for 2 hours at room temperature. Serum samples were serially diluted in 10% FBS in PBS and 100 μL was added to each well. The plates were then incubated for 2 hours at RT. HRP-conjugated anti-mouse IgG secondary antibody were diluted in 10% FBS in PBS at 1:5000. 100 μL was added to each well and plates were incubated for 2 hours at RT. Finally, 100 μL of OptEIA 3,3’,5,5’-Tetramethylbenzidine substrate was added. The reaction was stopped after 5-10 minutes with 50 μL of 1M sulphuric acid. The absorbance was read on Versa Max microplate reader at 450 nm. Before the initiation of each step, plates were washed five times with 0.05% TWEEN 20 in PBS, except following the blocking step.

### Data Analysis and Statistics

All data analysis and statistics were performed using R-based computational tools. All figures were generated using ggplot2.

Seroconversions were logarithm transformed and analysis of these were on a transformed scale. Normality assumptions were well-met on the transformed scale. Logistic regression was used to explore the relationship between vaccination history and seroconversions and to explore the association between D0 antibody titers and vaccination history.

Beta-diversity was calculated using a Bray-Curtis dissimilarity index. Permutational multivariate analysis of variance (PERMANOVA) was used to explore the relationship between microbiome beta-diversity and categorical variables. Logistic regression was used to explore the relationship between beta-diversity and vaccination history. The relationship of alpha diversity with categorical variables was explored using ANOVA.

Rank correlation (Spearman) was used to explore the strength of association between logarithm-transformed seroconversion and average daily fibre intake.

Comparisons between vaccine responses in mouse groups were made using Student’s t-test (two group comparison) or ANOVA (multiple group comparison).

## Results

### Influenza Vaccination History Strongly Influences Vaccine Responses

**Figure 1:**
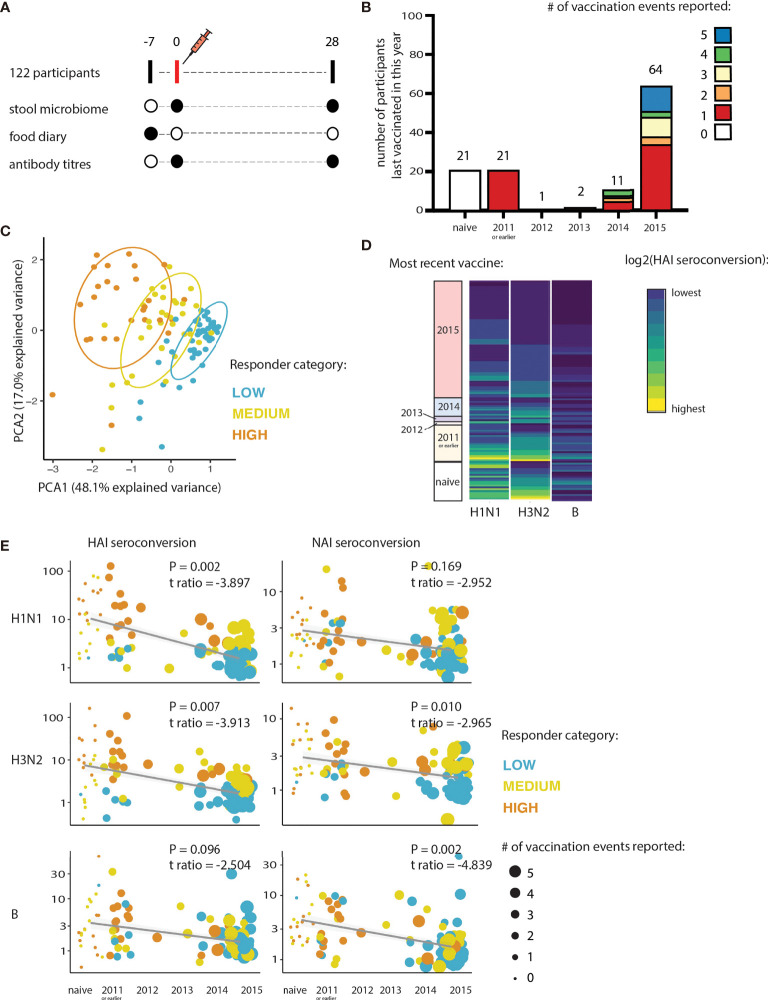
Influenza vaccine responses are determined by vaccination history. **(A)** Schematic overview of sampling timepoints relative to the vaccination event. **(B)** Histogram summarizes the number of seasonal-influenza vaccines received self-reported by participants at least 4 years prior to the study (2012-2015 inclusive; or from 2011-2015 inclusive). **(C)** Principal component analysis of both HAI and NAI seroconversion of each participant. Participants are coloured according to their responder category. **(D)** Heatmap showing relationship between seroconversion and year of most recent seasonal-influenza vaccines received by participants in the 4 years prior to the study (2012-2015), and 2011 or earlier. Hemagglutinin (HAI) seroconversion to each of the influenza subtypes contained in the vaccine is shown. **(E)** Relationship between seroconversion and vaccine history. Hemagglutinin (HAI) and Neuraminidase (NAI) seroconversions are shown, as is the response to each of the influenza subtypes contained in the vaccine.

122 healthy participants aged 18-64 received the 2016 trivalent influenza vaccine (TIV) as part of our study. Detailed study characteristics are described elsewhere (16). At the first visit, participants provided a food diary completed across the prior 7 days along with a stool sample collected between day 3 and 7 of diary completion (**Figure 1A**). In addition, an influenza vaccination history for 120 of the 122 participants was documented (**Figure 1B**). 21 of the participants (17.5%) had not received any previous influenza vaccination (naïve). Participants were stratified into low, medium, or high responders on the basis of their fold change in hemagglutinin (HAI)-specific antibodies to H1N1 and H3N2 pre-vaccination and 28 days post vaccination (seroconversion). These classifications broadly characterized the response to all three influenza strains in the vaccine (H1N1, H3N2, and B) for both HAI and neuraminidase (NAI) (**Figure 1C**).

We found that how recently participants received their last influenza vaccine had a significantly negative effect on the strength of the antibody response induced by vaccination (logistic regression; H1N1: t-ratio -9.538, P=2.49e-16; H3N2: t-ratio -6.988, P=1.78e-10; B: t-ratio -4.665, P=8.22e-06; **Figure 1D**). The frequency of vaccination (total number of vaccination events) also negatively impacted the strength of the antibody response to the 2016 TIV. The effect of vaccine history was consistent across the three influenza subtypes for both HAI and NAI seroconversion (logistic regression, **Figure 1E**). Vaccine history score was determined by combining both the frequency of previous vaccinations and the years since the most recent vaccination event (vaccination history score = frequency of previous vaccinations * years since most recent vaccine). There was no significant effect of vaccination history on the HAI or NAI baseline antibody titers (at Day 0) to the 2016 TIV-vaccine specific strains (**Supplementary Figure 2**; logistic regression, P>0.1). Because the B strain elicited a much weaker humoral response than the 2 influenza A strains, we focused on H1N1 and H3N2 for subsequent analysis.

### A Unique Microbiome Signature Is Correlated With the Vaccine Response to Each Influenza Strain

**Figure 2:**
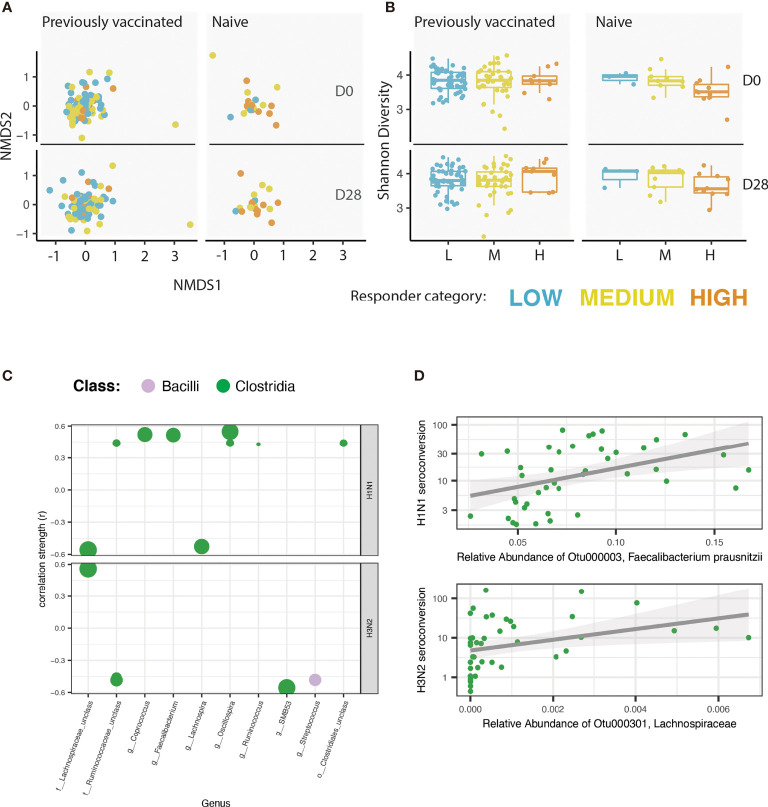
A unique microbiome signature is correlated with the vaccine response to each of the influenza strains. **(A)** Non-metric multidimensional scaling (NMDS) of the microbiome for each participant. NMDS is faceted by vaccination history (naïve *vs*. previously vaccinated) and by timepoint [pre-vaccine (D0) or 25 - 28 days post vaccine (D28)]. Colour of each point reflects the responder category of the participant. **(B)** Shannon index of alpha diversity. Plot is faceted by vaccination history (naïve *vs*. previously vaccinated) and by timepoint (pre-vaccine or 28 days post vaccine). Colour of each point reflects the responder category of the participant. **(C)** Correlation analysis showing the strength of Spearman correlation of operational taxonomic units (OTUs) with HAI seroconversion in individuals previously naïve to influenza vaccination. Each point is one OTU. Colour of each point represents the class level taxonomic assignment of each OTU. OTUs are organized (x-axis) by genus, or by the lowest level of taxonomic hierarchy determined. Size of each point corresponds to -log (P value). Only OTUs with a correlation coefficient > |0.15| and an adjusted P value < 0.05 are shown. Plot is faceted by influenza strain: H1N1 (top), H3N2 (bottom). **(D)** Scatter-plot showing the correlation between the OTU with the strongest correlation with H1N1 seroconversion (Faecalibacterium prausnitzii; top) and H3N2 seroconversion (identified to the family level as Lachnospiraceae; bottom).

To investigate the role of the intestinal microbiome in shaping antibody responses to seasonal influenza vaccine, we collected faecal samples prior to vaccination (D0), and again at a particular day, between the day 25-28 timepoint, post vaccination (D28). An overview of the microbiome of each participant at each timepoint is shown in **Supplementary Figure 3**. We found that vaccine history did not significantly alter beta diversity (logistic regression, P > 0.1, t-ratio 0.00553). Furthermore, there was no significant difference between the low, medium, or high vaccine responders in overall community composition (beta diversity, PERMANOVA, P >0.1) (**Figure 2A** **)**. Our investigation did not identify any significant difference in Shannon-index (alpha diversity) between the low, medium, and high vaccine responders (ANOVA, P >0.1). This was true at D0 and at D28, and independent of vaccine history (**Figure 2B**). However, we did find a unique microbiome signature at the operational taxonomic unit (OTU) level associated with responsiveness to each of the influenza subtypes. Across all participants, we found 12 OTUs correlated with H1N1 seroconversion (Spearman correlation, r > |0.15|, P_adj_< 0.05, **Supplementary Table 2.1** and **Supplementary Figure 4A** **)** and 13 OTUs correlated with H3N2 seroconversion (Spearman correlation, r > |0.15|, P_adj_ < 0.05, **Supplementary Table 2.2** and **Supplementary Figure 4B** **)**. We found stronger correlations between the microbiome and HAI seroconversion when we limited our analysis to individuals naïve to previous influenza vaccination. In naïve participants, we identified 10 OTUs that correlated with H1N1 seroconversion (Spearman correlation, r > |0.15|, P_adj_< 0.05, **Figures 2C, D**, **Supplementary Table 3.1** and **Supplementary Figure 5A** **)** and 5 OTUs correlated with H3N2 seroconversion (Spearman correlation, r > |0.15|, P_adj_ < 0.05, **Figures 2C, D**, **Supplementary Table 3.2** and **Supplementary Figure 5B** **).** The OTU most strongly associated with a H1N1 seroconversion, Faecalibacterium prausnitzii **(** **Figure 2C** **)**, is well studied for its ability to immunomodulate, and for its ability to ferment dietary fibres to SCFAs (22).

### Fibre Intake Positively Correlates With Vaccine Responses, Particularly in Individuals Previously Naïve to Influenza Vaccination

**Figure 3:**
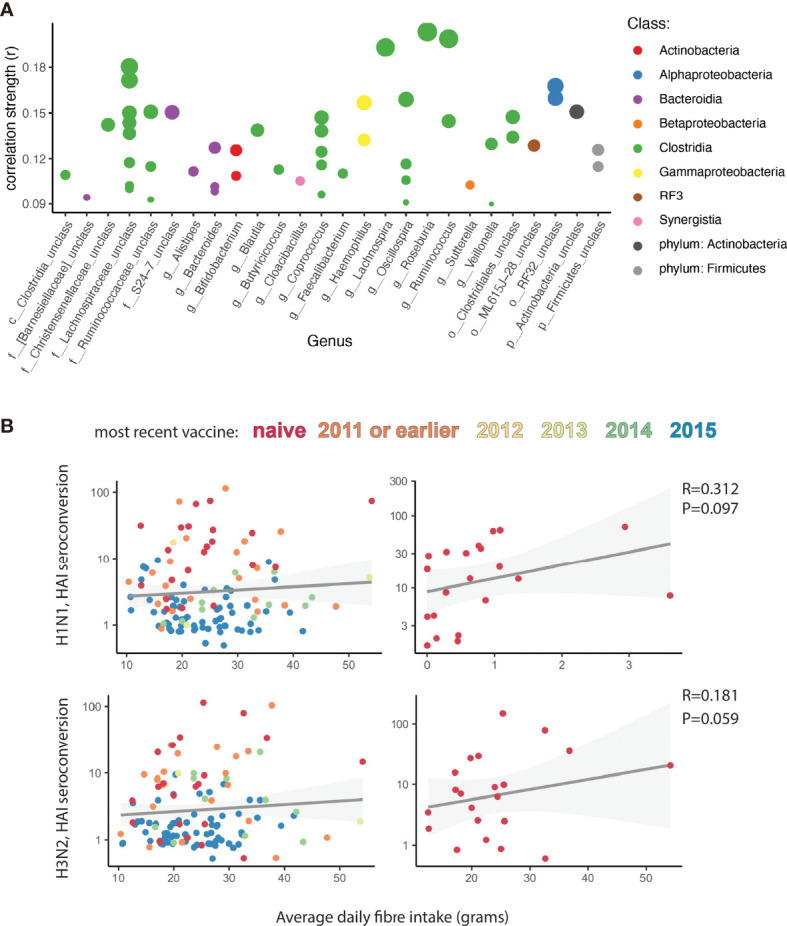
Fiber intake correlates with influenza vaccine responses. **(A)** Correlation analysis showing strength of Spearman correlation of operational taxonomic units (OTUs) with average dietary fibre intake. Each point is one OTU. Colour of each point represents the class level taxonomic assignment of each OTU. OTUs are organized (x-axis) by genus, or lowest level of taxonomic hierarchy determined. Size of each point corresponds to -log (P value). Only OTUs with an adjusted P value < 0.05 are shown. **(B)** Correlation analysis of average dietary fibre intake with Hemagglutinin (HAI) seroconversion. Colour represents the number of self-reported seasonal influenza vaccinations each participant received in the last 5 years. On the left all participants are analysed. On the right only participants naïve to previous influenza vaccination are analysed. Grey shadow represents 95% confidence interval.

To assess if diet had any impact on microbiota and TIV specific response, we analysed participants’ habitual dietary intake. Of particular interest to us was fibre intake, because of its known immunomodulatory properties (23). We first looked at microbial species that correlate with total dietary fibre intake and found 52 Operational Taxonomic Units (OTUs) that significantly increased with higher fibre intake (Spearman correlation, P_adj_ < 0.05, **Figure 3A**, **Supplementary Table 4** and **Supplementary Figure 6**). These OTUs were dominated by the class Clostridia, the class containing the majority of microbes with the ability to ferment indigestible fibre into SCFAs (**Figure 3A**) (24). As this class was disproportionately represented among the intestinal bacteria correlated with HAI seroconversion in vaccination naïve participants (14/15 = 93%), as compared to previously vaccinated participants (14/25 = 56%), we next looked at the inter-relationship between dietary fibre, antibody responses and vaccination history. Reflecting the marked preponderance of Clostridia within microbial classes associated with dietary fibre intake and *de novo* vaccine responses, we found the beneficial impact of dietary fibre on HAI seroconversion to be largely restricted to vaccination naïve individuals, albeit without reaching statistical significance in this under-powered subpopulation (Spearman correlation; H1N1 R =0.312, P=0.097; H3N2 R=0.181, P=0.059; **Figure 3B**).

In a more conservative analysis of the food diaries, of dietetic relevance, we excluded a) individual days in which participants reported an energy intake less than 1.2 X the Schofield predicted base metabolic rate and b) any participant that did not report at least 3 days with an energy intake higher than 1.2 X the Schofield predicted base metabolic rate. In both instances, similar trends were observed (**Supplementary Figure 7**).

### Dietary Fibre and Fibre Fermentation Is Required for Optimal Antibody Response in a Mouse Model of Influenza Vaccination

**Figure 4:**
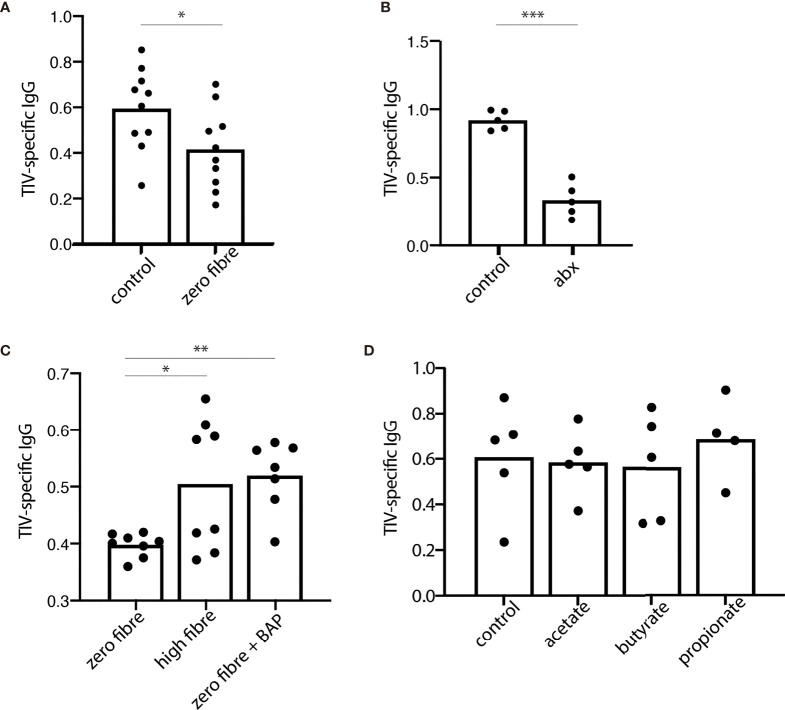
Dietary fibre and fibre fermentation are required for optimal antibody response in a mouse model of influenza vaccination. 28 days after receiving the trivalent influenza vaccine (TIV), serum levels of TIV-specific IgG were assessed from mice allocated to various fibre interventions. **(A)** TIV-specific IgG in the serum from mice fed a control or zero fibre diet. **(B)** TIV-specific IgG from mice on a control diet, with (abx) or without (control) the addition of an antibiotic cocktail to the drinking water. **(C)** TIV-specific IgG from mice on a zero-fibre diet, a high fibre diet, or a zero fibre diet supplemented with a cocktail of short chain fatty acids containing butyrate, acetate, and propionate **(D)** TIV-specific IgG from mice on a control diet supplemented with acetate, butyrate, or propionate. Data from **(A, C)** are from two independent experiments; samples from replicate groups are combined for analysis [**(A)** n=5/treatment/experiment **(B)** n=3-4/treatment/experiment]. Data from **(B, D)** are representative from 2 independent experiments (n = 5/treatment/experiment). Error bars in all panels show standard error of the mean. *P < 0.05. **P < 0.01. ***P < 0.001. BAP, butyrate, acetate, and propionate.

Dietary fibre is fermented by the healthy human microbiome into SCFAs, which have been well studied for their immuno-modulatory properties (25). We therefore wanted to further investigate the role of fibre and fibre fermentation products in antibody production in mice with no previous exposure to influenza or influenza vaccine, similarly to previous observations documenting the influence of fibre on antibody production at steady-state and in the context of infection (5). We found that mice on a fibre-free diet had an impaired IgG_total_, but not IgG1 antibody responses to TIV compared to those on a standard, high-fibre diet (**Figure 4A** and **Supplementary Figure 8A**). Next, we hypothesized that it was not the fibre itself, which was responsible for this phenotype, but rather the fibre fermentation products generated by the microbiota. To test this, we exposed mice to broad spectrum antibiotics to deplete their microbial fermentation capacity. Following three days of antibiotics, the frequencies of Firmicutes and Verrucomicrobia were diminished, while Actinobacteria and Bacteroidetes increased in frequency. After 10 days of antibiotics the faecal microbiota was further altered, with an expansion of Verrucomicrobia, Firmicutes and β-proteobacteria (**Supplementary Figure 9A** **)**. Consistent with the concept that microbial fibre fermentation products contributed to the effect, we found that mice on antibiotics had impaired IgG_total_ antibody responses to TIV compared to their normo-biotic counterparts, as previously reported (3) (**Figure 4B**). The deficit in the TIV-specific antibody response could be seen as early as 14 days post vaccination in both TIV-specific total IgG, and TIV-specific IgG1 (**Supplementary Figure 9B**). The effect of antibiotic treatment was specific to the TIV-antibody response, and not a broad effect of the antibiotics on humoral immunity (**Supplementary Figure 9C**). Further supporting the concept that microbial fermentation of dietary fibre to SCFAs contributed to the TIV-specific antibody response, we found that supplementation of the SCFAs butyrate, acetate, and propionate to mice on a fibre-free diet phenocopied mice on a high fibre diet (**Figure 4C** and **Supplementary Figure 8B**). In the presence of adequate dietary fibre (control diet), the addition of acetate, butyrate, or propionate had no further benefit to antibody responses (**Figure 4D** and **Supplementary Figure 8B**).

## Discussion

The efficacy of the seasonal influenza vaccine varies significantly from person to person (26). A strong antibody response is crucial for effective protection against influenza. In this study, we found that dietary fibre intake in human participants appear to be contributory to mounting a robust vaccine response.

This study provides insight on the impact of prior vaccination events to influenza vaccine humoral immunity. We observed an inverse correlation between previous vaccination events and seroconversion across the three influenza subtypes, for both HAI and NAI. This phenomenon has been observed previously with other trivalent inactivated influenza vaccines (27–35). Noticeably, the impact of both diet and of the microbiome was greatest in those subjects who self-reportedly never received the seasonal influenza vaccine before participating in this study. Similarly, Hagan et al. found that the impact of antibiotic exposure to humoral immunity using the same vaccine in a human trial was greatest in subjects with low baseline titers (7). The authors hypothesized that mechanisms involved in recall responses could be more resilient to changes in the gut microbiota than those involved in a primary response (7), a hypothesis congruent with our observations.

A number of clinical studies have attempted to identify the microbial and molecular determinants of influenza vaccine responsiveness, which is notoriously inconsistent throughout the general population (7–10). While these studies, alongside preclinical models (3–6), clearly demonstrate a microbiome-dependent effect, they have not provided actionable results. Importantly, our observations suggest that part of the microbial determinants of influenza vaccination responsiveness are secondary to dietary fibre intake -a finding which may generate new public health strategies to enhance population-based vaccination effectiveness.

Dietary fibre acts as the substrate for the microbial fermentation of SCFAs, which have been well studied as potent immune-modulatory metabolites capable of influencing cellular responses in locations beyond the gut. Systemic levels of SCFAs are most directly influenced by the amount of dietary fibre consumed (36). It has previously been observed in rats that dietary fibre intake potentiates IgA responses (37). SCFAs are the major dietary fibre metabolites responsible for augmenting antibody production in both mucosal tissues and systemically (5). Thus, our findings support the previously identified association of fibre consumption and antibody production.

Using a preclinical mouse model of TIV vaccination, we were able delineate the role of dietary fibre from SCFAs. We found that mice on a diet devoid of dietary fibre or mice lacking fermentation capacity (antibiotic treated) were inhibited in their antibody responses. Supplementing these mice with a mixture of SCFAs could restore antibody responses. Interestingly, SCFA administration together with high levels of dietary fibre did not further enhance antibody responses beyond a high fibre diet, indicating that SCFAs at supraphysiological concentrations have no added benefit in this context. Interestingly, while total vaccine-specific IgG levels were impaired in the absence of SCFAs, levels of vaccine-specific IgG1 were not. Future work should investigate how SCFA levels impact vaccine specific IgG2 and IgG3, as presumably one or both subclasses are reduced.

In addition to dietary fibre, another major determinant of SCFA levels is the presence of microbial species capable of performing the fermentation (38). Understanding the microbial ecology of this process across individuals in relation to their vaccine-responses may provide important insight into how to optimize vaccine efficacy. Future work should look at SCFA production pathways using shot-gun metagenomics (39, 40). Integrating data around the microbiome, diet, and systemic levels of SCFAs will provide a clearer picture of how these metabolites influence vaccine responses.

Vaccines represent one of the most widely used and important public health interventions. Despite this, we do not fully understand why responses to vaccines vary widely between individuals and across populations, and perhaps more importantly, whether similar microbial, molecular or dietary determinants underlie the effectiveness to immunologically distinct vaccine formulations (e.g. adjuvanted *vs* non-adjuvanted, protein *vs* mRNA), as the impact of SCFA may very well be vaccine-specific (41). In this study we present data to suggest that public health messages to increase dietary fibre intake could assist in more successful vaccine protection.

## Data Availability Statement

The raw data supporting the conclusions of this article will be made available by the authors, without undue reservation. The microbiome data related to this study are available at European Nucleotide Archive (http://www.ebi.ac.uk/ena) under study accession no PRJEB48578. Additional clinical data are available from the corresponding on request.

## Ethics Statement

The studies involving human participants were reviewed and approved by New Zealand Central Health and Disability Ethics Committee. The patients/participants provided their written informed consent to participate in this study.

## Author Contributions

HP, NS, IB, and EF-B provided clinical study conceptualization. AC and OG formulated and coordinated the overarching investigation process. AC, AM, HP, NS, AJ, AGe, KG, AGr, WY, DT, JS, TO, AT, SP, SE, GW, AP-S, JR, RB, MW, IB, and EF-B acquired, curated and/or analysed data. AC, AM, KG, AGr, DO’S, JT, and OG provided critical review, commentary and/or revision of the data. The manuscript was written by AC, DO’S, and OG and commented on by all authors. All authors contributed to the article and approved the submitted version.

## Funding

This work was supported by funding from the New Zealand Ministry for Business, Innovation and Employment (MBIE), as part of the High-Value Nutrition National Science Challenge, the independent research organization grant from the Health Research Council of New Zealand, and the Dines Family Charitable Trust, New Zealand.

## Conflict of Interest

SE and GW were employed by Food Savvy.

The remaining authors declare that the research was conducted in the absence of any commercial or financial relationship that could be construed as a potential conflict of interest.

## Publisher’s Note

All claims expressed in this article are solely those of the authors and do not necessarily represent those of their affiliated organizations, or those of the publisher, the editors and the reviewers. Any product that may be evaluated in this article, or claim that may be made by its manufacturer, is not guaranteed or endorsed by the publisher.
