# Supplementary material for: Potential Association Between Dietary Fibre and Humoral Response to the Seasonal Influenza Vaccine
Source: Front Immunol. 2021 Nov 17;12:765528. doi: 10.3389/fimmu.2021.765528 (PMC8635806; doi:10.3389/fimmu.2021.765528)
Supplement: Supplementary file 1 [file DataSheet_1.pdf]

Supplementary Table 1: Microbiome qPCR primers

TARGET	*PRIMER*	*SEQUENCE (5'- 3')*	*SUPPLIER*
BACTEROIDETES	Bac960F	GTTTAATTCGATGATACGCGAG	Sigma
	Bac1100R	TTAAGCCGACACCTCACGG	
FIRMICUTES	Firm934F	GGAGCATGTGGTTTAATTCGAAGCA	Sigma
	Firm1060R	AGCTGACGACAACCATGCAC	
ACTINOBACTERIA	Act664F	TGTAGCGGTGGAATGCGC	Sigma
	Act941R	AATTAAGCCACATGCTCCGCT	
CANDIDATUS SACCHARIBACTERIA	Sac1031F	AAGAGAACTGTGCCTTCGG	Sigma
	Sac1218R	GCGTAAGGGAAATACTGACC	
DEFERRIBACTERES	Defer1115F	CTATTTCCAGTTGCTAACGG	Sigma
	Defer1265R	GAGATGCTTCCCTCTGATTATG	
VERRUCOMICROBIA	Ver1165F	TCATGTCAGTATGGCCCTTAT	Sigma
	Ver1263R	CAGTTTTCAGGATTTCCTCCGCC	
TENERICUTES	Ten662F	ATGTGTAGCGGTAAAATGCGTAA	Sigma
	Ten862R	CATACTTGCGTACGTACTACT	
Β PROTEOBACTERIA	Beta979F	AACGCGAAAAACCTTACCTACC	Sigma
	Beta1130R	TGCCCTTTCGTAGCAACTAGTG	
Ε PROTEOBACTERIA	Epsilon940F	TAGGCTTGACATTGATAGAATC	Sigma
	Epsilon1129R	CTTACGAAGGCAGTCTCCTTA	
Γ PROTEOBACTERIA	Gamma877F	GCTAACGCATTAAGTACCCCG	Sigma
	Gamma1066R	GCCATGCAGCACCTGTCT	
UNIVERSAL 16S	926F	AAACTCAAATGAATTGACGG	Sigma
	1062R	CTCACAACACGAGCTGAC	

Supplementary table 2.1: Operational taxonomic units (OTUs) with a correlation coefficient > |0.15| significantly correlated with H1N1 seroconversion in all participants.

OTU identifier	*P-value*	*Correlation coefficient (r)*	*Benjamini-Hochberg P-value*	*Phylum*	*Class*	*Order*	*Family*	*Genus*	*Species*
Otu000004	0.0046	0.1699	0.0069	p__Bacteroidetes	c__Bacteroidia	o__Bacteroidales	f__Porphyromonadaceae	g__Parabacteroides	s__distasonis
Otu000034	0.0126	-0.1569	0.0147	p__Firmicutes	c__Clostridia	o__Clostridiales	f__Veillonellaceae	g__Phascolarctobacterium	g__Phascolarctobacterium_unclass
Otu000105	0.0006	0.2257	0.0021	p__Proteobacteria	c__Betaproteobacteria	o__Burkholderiales	f__Alcaligenaceae	g__Sutterella	g__Sutterella_unclass
Otu000131	0.0067	0.1680	0.0081	p__Firmicutes	c__Clostridia	o__Clostridiales	f__Lachnospiraceae	f__Lachnospiraceae_unclass	f__Lachnospiraceae_unclass
Otu000144	0.0054	-0.1719	0.0121	p__Firmicutes	c__Bacilli	o__Turicibacterales	f__Turicibacteraceae	g__Turicibacter	g__Turicibacter_unclass
Otu000161	0.0000	-0.2263	0.0003	p__Firmicutes	c__Clostridia	o__Clostridiales	f__Lachnospiraceae	f__Lachnospiraceae_unclass	f__Lachnospiraceae_unclass
Otu000188	0.0007	0.2146	0.0021	p__Proteobacteria	c__Alphaproteobacteria	o__RF32	o__RF32_unclass	o__RF32_unclass	o__RF32_unclass
Otu000233	0.0170	-0.1501	0.0170	p__Bacteroidetes	c__Bacteroidia	o__Bacteroidales	f__[Odoribacteraceae]	g__Butyricimonas	g__Butyricimonas_unclass
Otu000310	0.0025	-0.1802	0.0113	p__Firmicutes	c__Clostridia	o__Clostridiales	f__Ruminococcaceae	g__Oscillospira	g__Oscillospira_unclass
Otu000451	0.0129	-0.1524	0.0147	p__Firmicutes	c__Clostridia	o__Clostridiales	f__Lachnospiraceae	f__Lachnospiraceae_unclass	f__Lachnospiraceae_unclass
Otu000480	0.0177	0.1610	0.0177	p__Firmicutes	c__Clostridia	o__Clostridiales	f__Ruminococcaceae	g__Oscillospira	g__Oscillospira_unclass
Otu000837	0.0131	-0.1511	0.0147	p__Firmicutes	c__Clostridia	c__Clostridia_unclass	c__Clostridia_unclass	c__Clostridia_unclass	c__Clostridia_unclass

Supplementary table 2.2: Operational taxonomic units (OTUs) with a correlation coefficient > |0.15| significantly correlated with H3N2 seroconversion in all participants.

OTU identifier	*P-value*	*Correlation coefficient (r)*	*Benjamini-Hochberg P-value*	*Phylum*	*Class*	*Order*	*Family*	*Genus*	*Species*
Otu000032	0.0193	-0.1601	0.0193	p__Proteobacteria	c__Gammaproteobacteria	o__Pasteurellales	f__Pasteurellaceae	g__Haemophilus	s__parainfluenzae
Otu000066	0.0018	-0.2020	0.0060	p__Firmicutes	c__Clostridia	o__Clostridiales	f__Lachnospiraceae	g__Coprococcus	g__Coprococcus_unclass
Otu000082	0.0113	0.1715	0.0198	p__Firmicutes	c__Clostridia	o__Clostridiales	f__Lachnospiraceae	f__Lachnospiraceae_unclass	f__Lachnospiraceae_unclass
Otu000083	0.0010	-0.1986	0.0049	p__Firmicutes	c__Bacilli	o__Lactobacillales	f__Streptococcaceae	g__Streptococcus	g__Streptococcus_unclass
Otu000085	0.0188	0.1534	0.0219	p__Bacteroidetes	c__Bacteroidia	o__Bacteroidales	f__Prevotellaceae	g__Prevotella	s__copri
Otu000126	0.0321	0.1513	0.0321	p__Firmicutes	c__Clostridia	o__Clostridiales	f__Ruminococcaceae	g__Ruminococcus	g__Ruminococcus_unclass
Otu000144	0.0036	-0.2005	0.0073	p__Firmicutes	c__Bacilli	o__Turicibacterales	f__Turicibacteraceae	g__Turicibacter	g__Turicibacter_unclass
Otu000151	0.0071	-0.1609	0.0118	p__Firmicutes	c__Clostridia	o__Clostridiales	f__Clostridiaceae	g__Clostridium	g__Clostridium_unclass
Otu000320	0.0037	0.1668	0.0128	p__Firmicutes	c__Clostridia	o__Clostridiales	f__Ruminococcaceae	f__Ruminococcaceae_unclass	f__Ruminococcaceae_unclass
Otu000414	0.0029	0.1728	0.0128	p__Actinobacteria	c__Actinobacteria	o__Bifidobacteriales	f__Bifidobacteriaceae	g__Bifidobacterium	s__bifidum
Otu000704	0.0149	0.1669	0.0209	p__Cyanobacteria	c__4C0d-2	o__YS2	o__YS2_unclass	o__YS2_unclass	o__YS2_unclass
Otu000837	0.0139	-0.1577	0.0174	p__Firmicutes	c__Clostridia	c__Clostridia_unclass	c__Clostridia_unclass	c__Clostridia_unclass	c__Clostridia_unclass
Otu001469	0.0093	0.1518	0.0198	p__Firmicutes	c__Clostridia	o__Clostridiales	o__Clostridiales_unclass	o__Clostridiales_unclass	o__Clostridiales_unclass

Supplementary table 3.1: Operational taxonomic units (OTUs) with a correlation coefficient > |0.15| significantly correlated with H1N1 seroconversion in participants naive to previous influenza vaccination.

OTU identifier	***P-value***	***Correlation coefficient (r)***	***Benjamini-Hochberg P-value***	***Phylum***	***Class***	***Order***	***Family***	***Genus***	***Species***
Otu000003	0.0005	0.5158	0.0101	p__Firmicutes	c__Clostridia	o__Clostridiales	f__Ruminococcaceae	g__Faecalibacterium	s__prausnitzii
Otu000059	0.0004	0.5212	0.0101	p__Firmicutes	c__Clostridia	o__Clostridiales	f__Lachnospiraceae	g__Coprococcus	s__eutactus
Otu000134	0.0003	-0.5264	0.0156	p__Firmicutes	c__Clostridia	o__Clostridiales	f__Lachnospiraceae	g__Lachnospira	g__Lachnospira_unclass
Otu000288	0.0047	0.4276	0.0379	p__Firmicutes	c__Clostridia	o__Clostridiales	f__Ruminococcaceae	g__Ruminococcus	g__Ruminococcus_unclass
Otu000404	0.0036	0.4396	0.0361	p__Firmicutes	c__Clostridia	o__Clostridiales	f__Ruminococcaceae	f__Ruminococcaceae_unclass	f__Ruminococcaceae_unclass
Otu000435	0.0035	0.4403	0.0361	p__Firmicutes	c__Clostridia	o__Clostridiales	f__Ruminococcaceae	g__Oscillospira	g__Oscillospira_unclass
Otu000511	0.0040	0.4354	0.0361	p__Firmicutes	c__Clostridia	o__Clostridiales	f__Ruminococcaceae	f__Ruminococcaceae_unclass	f__Ruminococcaceae_unclass
Otu000545	0.0002	0.5488	0.0101	p__Firmicutes	c__Clostridia	o__Clostridiales	f__Ruminococcaceae	g__Oscillospira	g__Oscillospira_unclass
Otu000689	0.0001	-0.5588	0.0109	p__Firmicutes	c__Clostridia	o__Clostridiales	f__Lachnospiraceae	f__Lachnospiraceae_unclass	f__Lachnospiraceae_unclass
Otu000702	0.0037	0.4388	0.0361	p__Firmicutes	c__Clostridia	o__Clostridiales	o__Clostridiales_unclass	o__Clostridiales_unclass	o__Clostridiales_unclass

Supplementary table 3.2: Operational taxonomic units (OTUs) with a correlation coefficient > |0.15| significantly correlated with H3N2 seroconversion in participants naive to previous influenza vaccination.

OTU identifier	***P-value***	***Correlation coefficient (r)***	***Benjamini-Hochberg P-value***	***Phylum***	***Class***	***Order***	***Family***	***Genus***	***Species***
Otu000046	0.0011	-0.4854	0.0353	p__Firmicutes	c__Clostridia	o__Clostridiales	f__Ruminococcaceae	f__Ruminococcaceae_unclass	f__Ruminococcaceae_unclass
Otu000083	0.0012	-0.4828	0.0353	p__Firmicutes	c__Bacilli	o__Lactobacillales	f__Streptococcaceae	g__Streptococcus	g__Streptococcus_unclass
Otu000213	0.0001	-0.5562	0.0115	p__Firmicutes	c__Clostridia	o__Clostridiales	f__Clostridiaceae	g__SMB53	g__SMB53_unclass
Otu000301	0.0001	0.5594	0.0086	p__Firmicutes	c__Clostridia	o__Clostridiales	f__Lachnospiraceae	f__Lachnospiraceae_unclass	f__Lachnospiraceae_unclass
Otu000391	0.0021	-0.4607	0.0471	p__Firmicutes	c__Clostridia	o__Clostridiales	f__Ruminococcaceae	f__Ruminococcaceae_unclass	f__Ruminococcaceae_unclass

Supplementary table 4: Operational taxonomic units (OTUs) significantly correlated with average daily fibre consumption.

OTU identifier	*P-value*	*Correlation coefficient (r)*	*Benjamini-Hochberg P-value*	*Phylum*	*Class*	*Order*	*Family*	*Genus*	*Species*
Otu000003	0.015625504	0.109389164	0.021635313	p__Firmicutes	c__Clostridia	o__Clostridiales	f__Ruminococcaceae	g__Faecalibacterium	s__prausnitzii
Otu000008	0.024715834	0.101660485	0.029659001	p__Bacteroidetes	c__Bacteroidia	o__Bacteroidales	f__Bacteroidaceae	g__Bacteroides	g__Bacteroides_unclass
Otu000013	0.029525775	0.098533586	0.032538609	p__Bacteroidetes	c__Bacteroidia	o__Bacteroidales	f__Bacteroidaceae	g__Bacteroides	g__Bacteroides_unclass
Otu000029	0.000149522	0.170824338	0.001614838	p__Firmicutes	c__Clostridia	o__Clostridiales	f__Lachnospiraceae	f__Lachnospiraceae_unclass	f__Lachnospiraceae_unclass
Otu000032	0.000523012	0.15645324	0.003138071	p__Proteobacteria	c__Gammaproteobacteria	o__Pasteurellales	f__Pasteurellaceae	g__Haemophilus	s__parainfluenzae
Otu000042	6.92E-05	0.179126793	0.000933803	p__Firmicutes	c__Clostridia	o__Clostridiales	f__Lachnospiraceae	f__Lachnospiraceae_unclass	f__Lachnospiraceae_unclass
Otu000065	0.012991538	0.112377255	0.019487308	p__Firmicutes	c__Clostridia	o__Clostridiales	f__Ruminococcaceae	g__Butyricicoccus	s__pullicaecorum
Otu000066	0.005931631	0.124391135	0.010676936	p__Firmicutes	c__Clostridia	o__Clostridiales	f__Lachnospiraceae	g__Coprococcus	g__Coprococcus_unclass
Otu000072	1.82E-05	0.192662962	0.000328395	p__Firmicutes	c__Clostridia	o__Clostridiales	f__Lachnospiraceae	g__Lachnospira	g__Lachnospira_unclass
Otu000075	0.004030028	0.12996219	0.00870486	p__Firmicutes	c__Clostridia	o__Clostridiales	f__Veillonellaceae	g__Veillonella	s__dispar
Otu000081	0.03450295	0.095728428	0.037263186	p__Firmicutes	c__Clostridia	o__Clostridiales	f__Lachnospiraceae	g__Coprococcus	g__Coprococcus_unclass
Otu000098	0.000857074	0.150434436	0.003689725	p__Bacteroidetes	c__Bacteroidia	o__Bacteroidales	f__S24-7	f__S24-7_unclass	f__S24-7_unclass
Otu000101	0.000198718	0.167661792	0.001788459	p__Proteobacteria	c__Alphaproteobacteria	o__RF32	o__RF32_unclass	o__RF32_unclass	o__RF32_unclass
Otu000105	0.023153756	0.102789454	0.02907681	p__Proteobacteria	c__Betaproteobacteria	o__Burkholderiales	f__Alcaligenaceae	g__Sutterella	g__Sutterella_unclass
Otu000116	0.005423281	0.125701536	0.010098523	p__Actinobacteria	c__Actinobacteria	o__Bifidobacteriales	f__Bifidobacteriaceae	g__Bifidobacterium	s__longum
Otu000141	6.13E-06	0.203082065	0.000273246	p__Firmicutes	c__Clostridia	o__Clostridiales	f__Lachnospiraceae	g__Roseburia	g__Roseburia_unclass
Otu000143	0.025277619	0.101269509	0.029673726	p__Firmicutes	c__Clostridia	o__Clostridiales	f__Lachnospiraceae	f__Lachnospiraceae_unclass	f__Lachnospiraceae_unclass
Otu000153	0.000888267	0.149990207	0.003689725	p__Firmicutes	c__Clostridia	o__Clostridiales	f__Lachnospiraceae	f__Lachnospiraceae_unclass	f__Lachnospiraceae_unclass
Otu000155	0.004413376	0.128671356	0.009166243	p__Tenericutes	c__RF3	o__ML615J-28	o__ML615J-28_unclass	o__ML615J-28_unclass	o__ML615J-28_unclass
Otu000175	0.003004784	0.134058015	0.007375378	p__Firmicutes	c__Clostridia	o__Clostridiales	o__Clostridiales_unclass	o__Clostridiales_unclass	o__Clostridiales_unclass
Otu000183	0.000824656	0.150912234	0.003689725	p__Firmicutes	c__Clostridia	o__Clostridiales	f__Ruminococcaceae	f__Ruminococcaceae_unclass	f__Ruminococcaceae_unclass
Otu000187	0.016228683	0.108767757	0.021908722	p__Actinobacteria	c__Actinobacteria	o__Bifidobacteriales	f__Bifidobacteriaceae	g__Bifidobacterium	g__Bifidobacterium_unclass
Otu000188	0.000397646	0.159701985	0.002784856	p__Proteobacteria	c__Alphaproteobacteria	o__RF32	o__RF32_unclass	o__RF32_unclass	o__RF32_unclass
Otu000193	0.002490182	0.136621973	0.006403326	p__Firmicutes	c__Clostridia	o__Clostridiales	f__Lachnospiraceae	f__Lachnospiraceae_unclass	f__Lachnospiraceae_unclass
Otu000230	0.047429628	0.089789929	0.047429628	p__Firmicutes	c__Clostridia	o__Clostridiales	f__Veillonellaceae	g__Veillonella	s__dispar
Otu000241	0.024028443	0.102149456	0.029489452	p__Firmicutes	c__Clostridia	o__Clostridiales	f__Lachnospiraceae	f__Lachnospiraceae_unclass	f__Lachnospiraceae_unclass
Otu000252	0.002119389	0.138788998	0.006023526	p__Firmicutes	c__Clostridia	o__Clostridiales	f__Lachnospiraceae	g__Blautia	s__producta
Otu000255	0.009391818	0.117475021	0.016359942	p__Firmicutes	c__Clostridia	o__Clostridiales	f__Lachnospiraceae	f__Lachnospiraceae_unclass	f__Lachnospiraceae_unclass

Otu000276	0.001351215	0.144684808	0.004560352	p__Firmicutes	c__Clostridia	o__Clostridiales	f__Ruminococcaceae	g__Ruminococcus	g__Ruminococcus_unclass
Otu000281	0.011059309	0.114930862	0.017062935	p__Firmicutes	p__Firmicutes_unclass	p__Firmicutes_unclass	p__Firmicutes_unclass	p__Firmicutes_unclass	p__Firmicutes_unclass
Otu000283	0.019263231	0.105918269	0.025371085	p__Firmicutes	c__Clostridia	o__Clostridiales	f__Ruminococcaceae	g__Oscillospira	g__Oscillospira_unclass
Otu000316	0.001469576	0.143601496	0.004668065	p__Firmicutes	c__Clostridia	o__Clostridiales	f__Lachnospiraceae	f__Lachnospiraceae_unclass	f__Lachnospiraceae_unclass
Otu000351	0.013618372	0.111620818	0.019875462	p__Bacteroidetes	c__Bacteroidia	o__Bacteroidales	f__Rikenellaceae	g__Alistipes	s__indistinctus
Otu000353	0.0048713	0.127256055	0.0097426	p__Bacteroidetes	c__Bacteroidia	o__Bacteroidales	f__Bacteroidaceae	g__Bacteroides	g__Bacteroides_unclass
Otu000371	0.01031724	0.116017805	0.016882756	p__Firmicutes	c__Clostridia	o__Clostridiales	f__Lachnospiraceae	g__Coprococcus	g__Coprococcus_unclass
Otu000376	0.005412421	0.125730719	0.010098523	p__Firmicutes	p__Firmicutes_unclass	p__Firmicutes_unclass	p__Firmicutes_unclass	p__Firmicutes_unclass	p__Firmicutes_unclass
Otu000392	0.026924853	0.100164811	0.030934937	p__Firmicutes	c__Clostridia	o__Clostridiales	f__Lachnospiraceae	f__Lachnospiraceae_unclass	f__Lachnospiraceae_unclass
Otu000435	0.010134552	0.116296012	0.016882756	p__Firmicutes	c__Clostridia	o__Clostridiales	f__Ruminococcaceae	g__Oscillospira	g__Oscillospira_unclass
Otu000436	0.001090429	0.14741852	0.004070845	p__Firmicutes	c__Clostridia	o__Clostridiales	o__Clostridiales_unclass	o__Clostridiales_unclass	o__Clostridiales_unclass
Otu000450	0.003415737	0.132283248	0.008019557	p__Proteobacteria	c__Gammaproteobacteria	o__Pasteurellales	f__Pasteurellaceae	g__Haemophilus	s__parainfluenzae
Otu000469	1.01E-05	0.198358606	0.000273246	p__Firmicutes	c__Clostridia	o__Clostridiales	f__Ruminococcaceae	g__Ruminococcus	g__Ruminococcus_unclass
Otu000472	0.001651683	0.142081979	0.004955049	p__Firmicutes	c__Clostridia	o__Clostridiales	f__Christensenellaceae	f__Christensenellaceae_unclass	f__Christensenellaceae_unclass
Otu000509	0.000836186	0.150740335	0.003689725	p__Actinobacteria	p__Actinobacteria_unclass	p__Actinobacteria_unclass	p__Actinobacteria_unclass	p__Actinobacteria_unclass	p__Actinobacteria_unclass
Otu000545	0.000412571	0.159268759	0.002784856	p__Firmicutes	c__Clostridia	o__Clostridiales	f__Ruminococcaceae	g__Oscillospira	g__Oscillospira_unclass
Otu000547	0.040363894	0.092836977	0.041916352	p__Firmicutes	c__Clostridia	o__Clostridiales	f__Ruminococcaceae	f__Ruminococcaceae_unclass	f__Ruminococcaceae_unclass
Otu000561	0.044773223	0.090887599	0.045618001	p__Firmicutes	c__Clostridia	o__Clostridiales	f__Ruminococcaceae	g__Oscillospira	g__Oscillospira_unclass
Otu000567	0.037147445	0.094375945	0.039332589	p__Bacteroidetes	c__Bacteroidia	o__Bacteroidales	f__[Barnesiellaceae]	f__[Barnesiellaceae]_unclass	f__[Barnesiellaceae]_unclass
Otu000803	0.019800641	0.105455044	0.025457967	p__Synergistetes	c__Synergistia	o__Synergistales	f__Synergistaceae	g__Cloacibacillus	g__Cloacibacillus_unclass
Otu000863	0.00113079	0.146958417	0.004070845	p__Firmicutes	c__Clostridia	o__Clostridiales	f__Lachnospiraceae	g__Coprococcus	g__Coprococcus_unclass
Otu000867	0.002234176	0.138083404	0.006032275	p__Firmicutes	c__Clostridia	o__Clostridiales	f__Lachnospiraceae	g__Coprococcus	g__Coprococcus_unclass
Otu001355	0.010991304	0.115027737	0.017062935	p__Firmicutes	c__Clostridia	o__Clostridiales	f__Ruminococcaceae	f__Ruminococcaceae_unclass	f__Ruminococcaceae_unclass
Otu002271	0.015595792	0.109420315	0.021635313	p__Firmicutes	c__Clostridia	c__Clostridia_unclass	c__Clostridia_unclass	c__Clostridia_unclass	c__Clostridia_unclass
