# Supplementary material for: Potential Association Between Dietary Fibre and Humoral Response to the Seasonal Influenza Vaccine
Source: Front Immunol. 2021 Nov 17;12:765528. doi: 10.3389/fimmu.2021.765528 (PMC8635806; doi:10.3389/fimmu.2021.765528)
Supplement: Supplementary file 2 [file DataSheet_2.zip › revision3_Supplementary figures.docx]

**
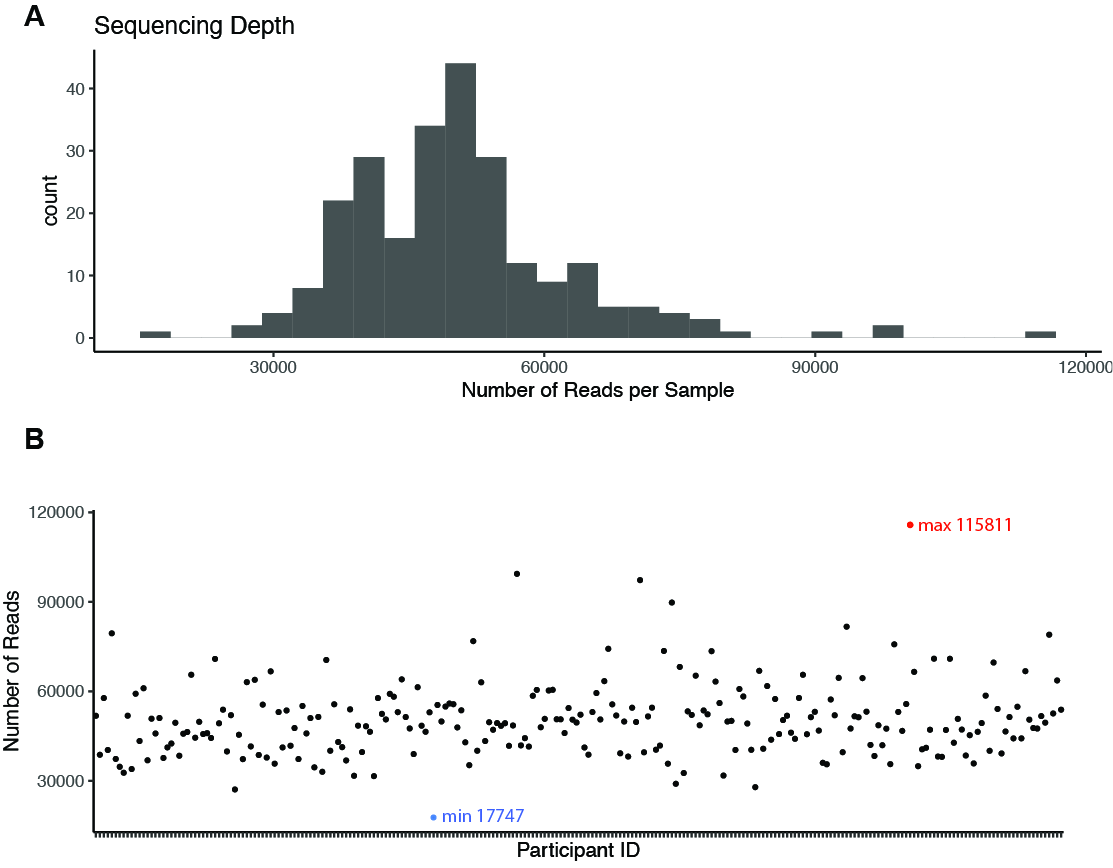
**

**Supplementary Figure 1:** Sequencing depth after processing raw reads to operational taxonomic units (OTUs). (A) Histogram showing distribution of the number of reads per sample**.** (B) Number of reads in each sample with the lowest and highest samples highlighted.


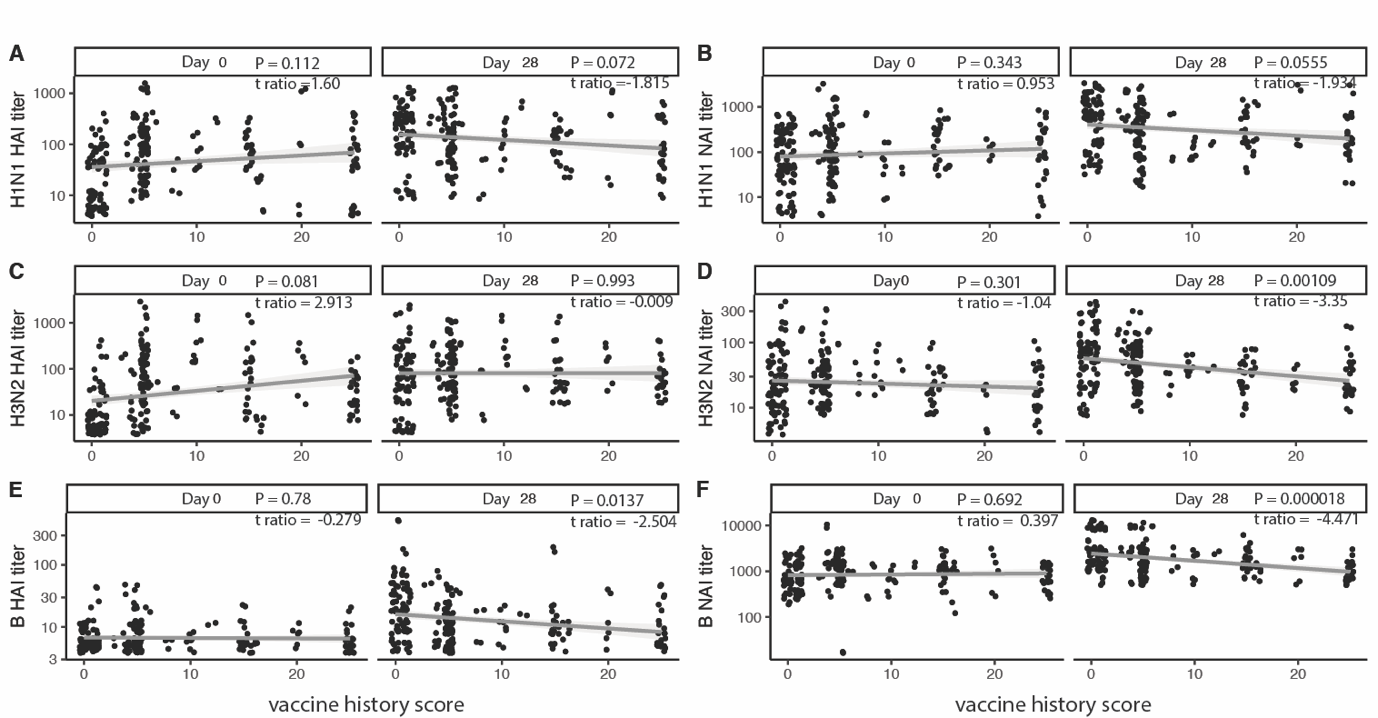


**Supplementary Figure 2**: **Relationship between influenza subtype-specific antibody titres and vaccination history score**. Y axis; antibody titres. X axis; vaccine history. Vaccine history score was determined by combining both the frequency of previous vaccinations and the years since the most recent vaccination event (vaccination history score = frequency of previous vaccinations * years since most recent vaccine). Hemagglutinin (HAI) and Neuraminidase (NAI) specific antibody titres are shown, as is the response to each of the influenza subtypes contained in the vaccine. Each plot is faceted by timepoint (day 0 and 28 days post vaccination). (A) Influenza A H1N1 HAI titres (B) Influenza A H1N1 NAI titres (C) Influenza A H3N2 HAI titres (D) Influenza A H3N2 NAI titires (E ) Influenza B HAI titres (F) Influenza B titres.


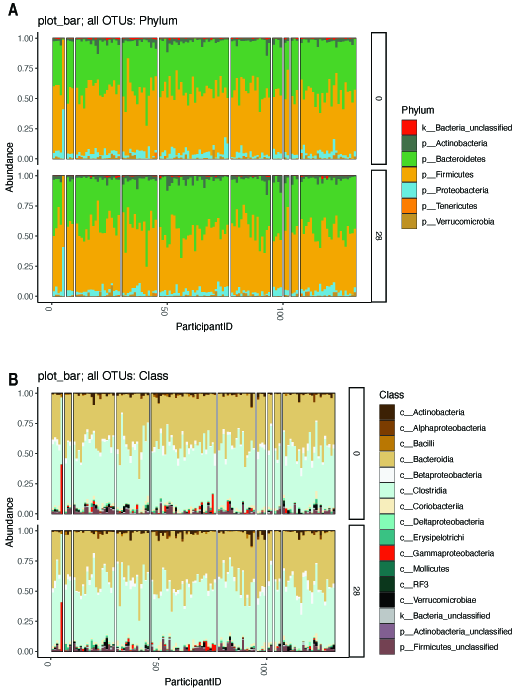


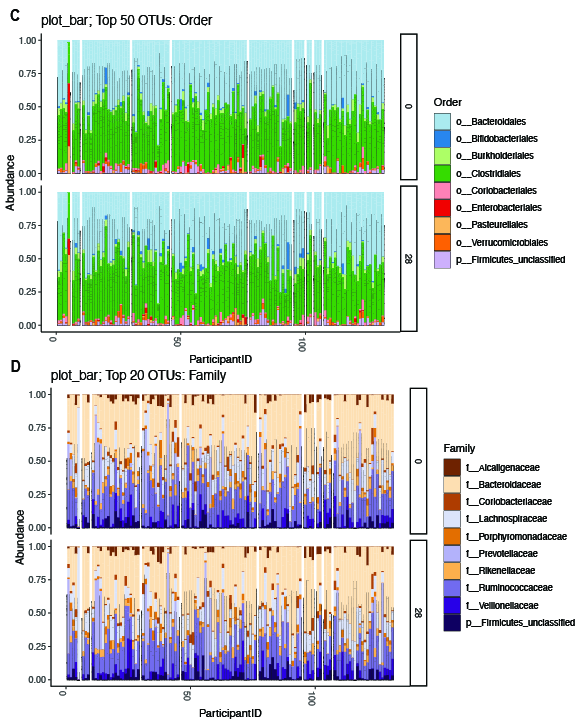


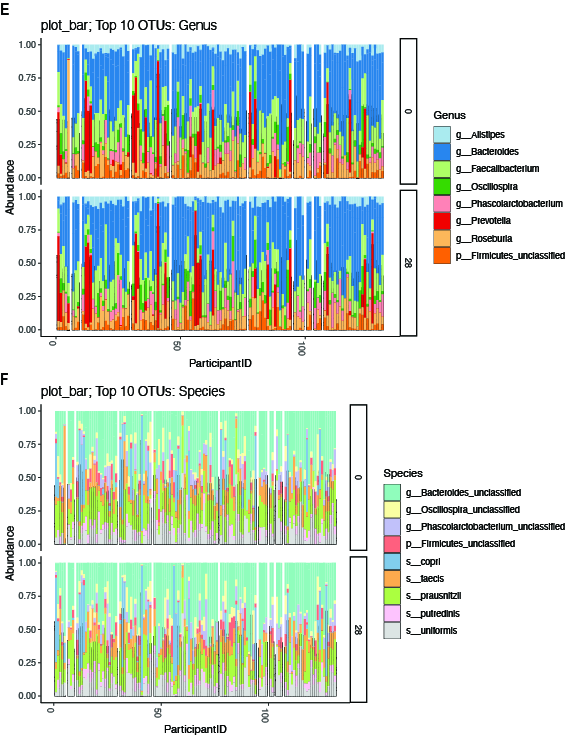


**Supplementary Figure 3**: **Compositional data of the microbiome for each participant at each timepoint**. Y axis; relative abundance of each OTU, organized by taxonomic assignment. (A) All OTUs are shown, colour represents phylum. (B) All OTUs are shown, colour represents class. (C) Top 50 most abundant OTUs are shown, colour represents class. (D) Top 20 most abundant OTUs are shown, colour represents order. (E ) Top 10 most abundant OTUs are shown, colour represents genus. (F) Top 10 most abundant OTUs are shown, colour represents species. Each plot is faceted by timepoint (day 0 and 28 days post vaccination). OTU = operational taxonomic unit.

**A**


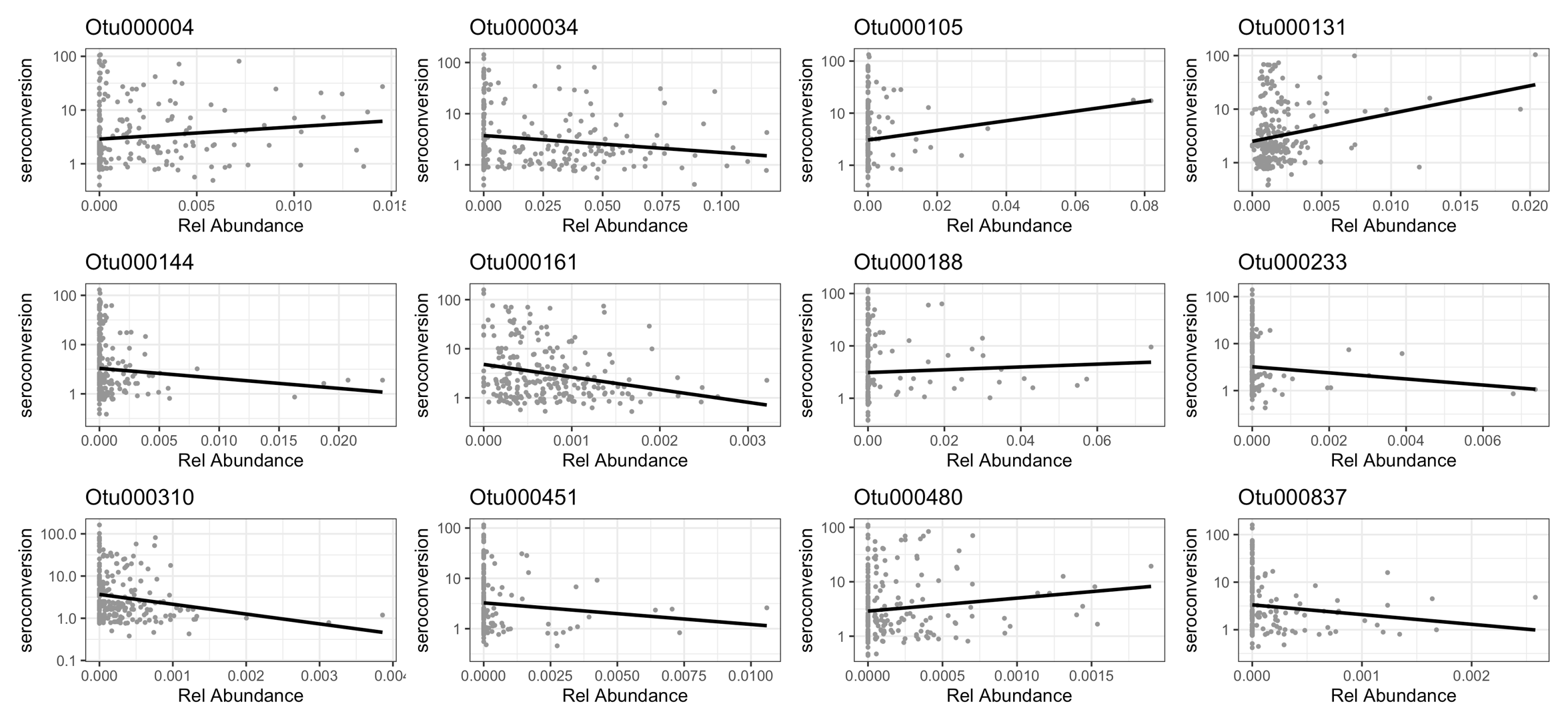


B


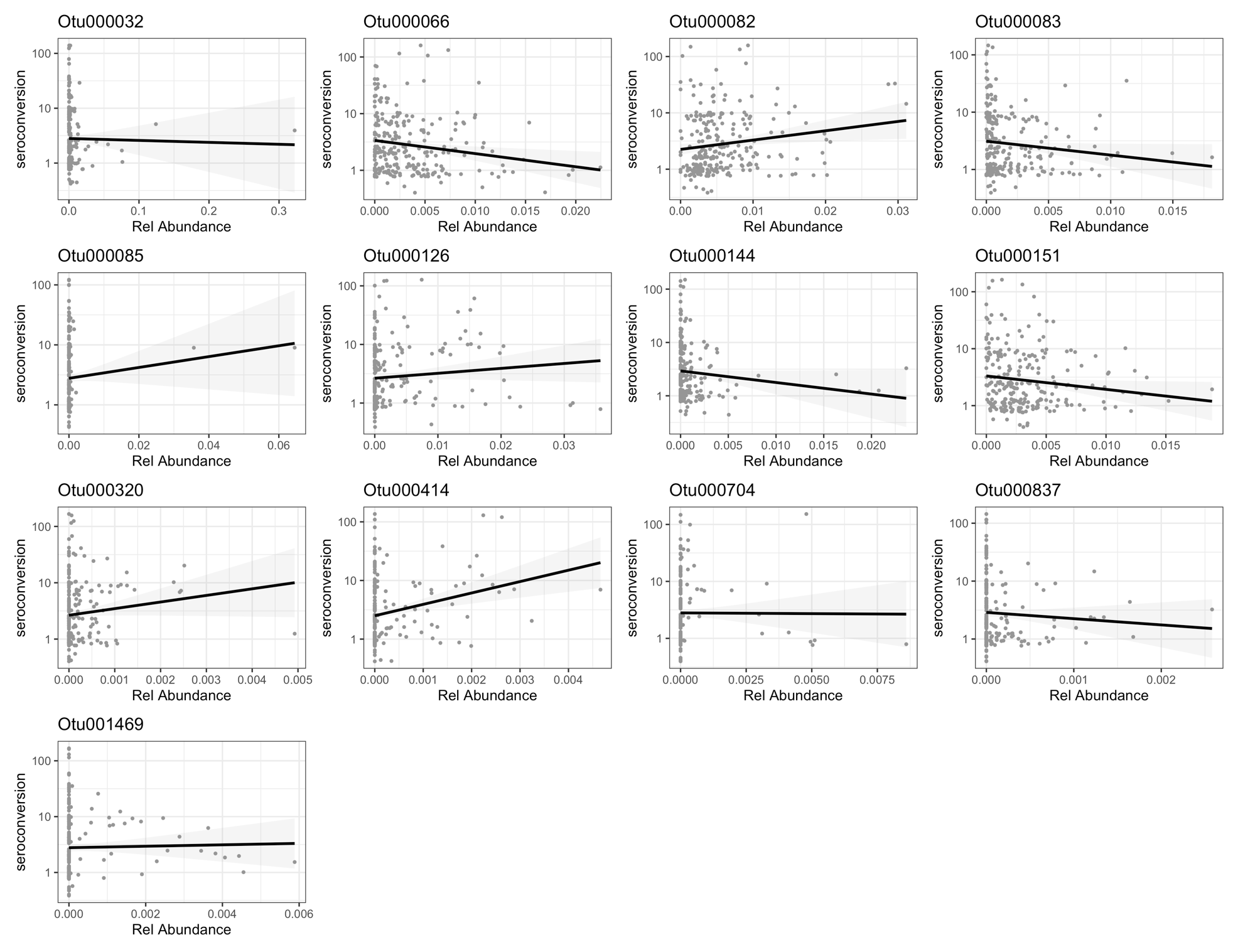


**Supplementary Figure 4:** Scatter plots of operational taxonomic units (OTUs) with a correlation coefficient > |0.15| that are significantly correlated with (A) H1N1 and (B) H3N2 seroconversion in all participants. Rel = relative.

A


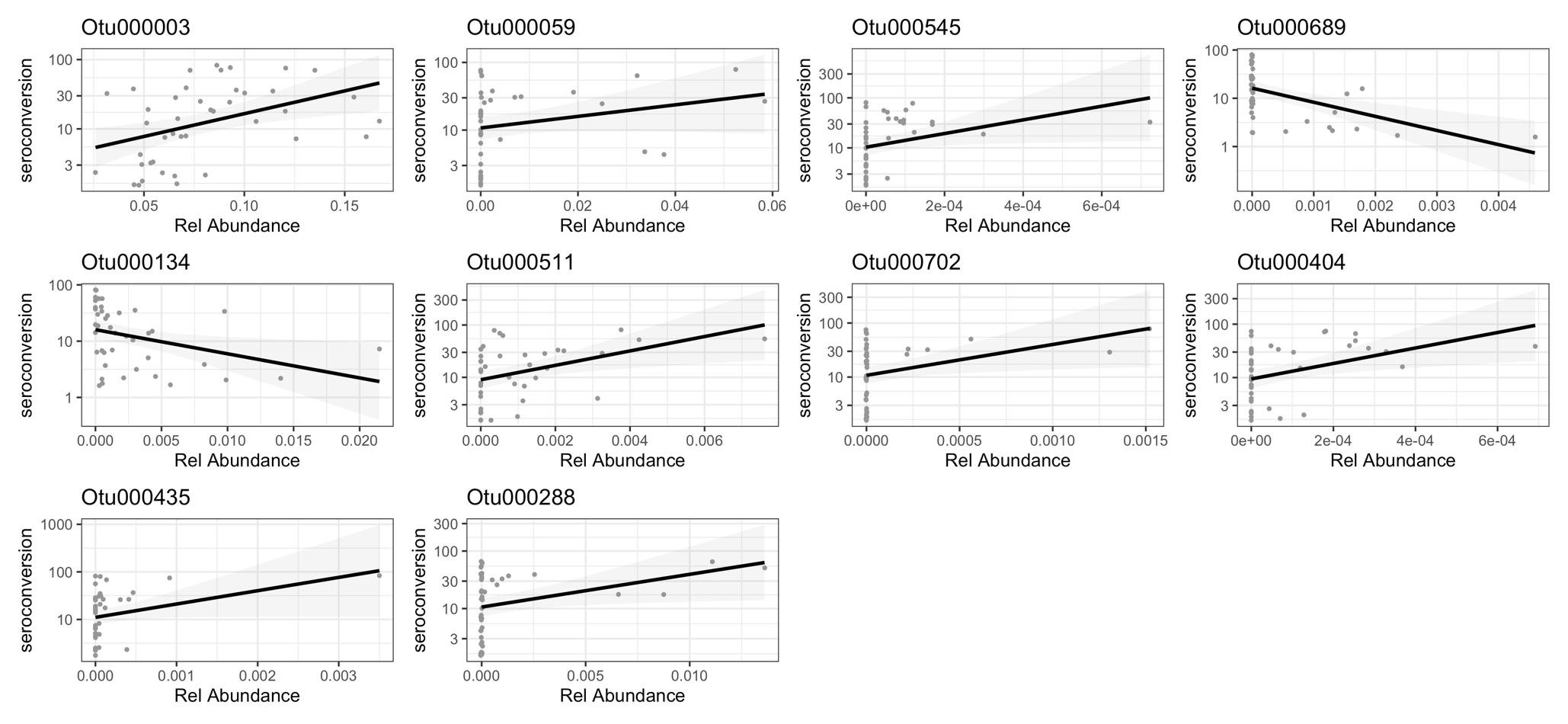


B


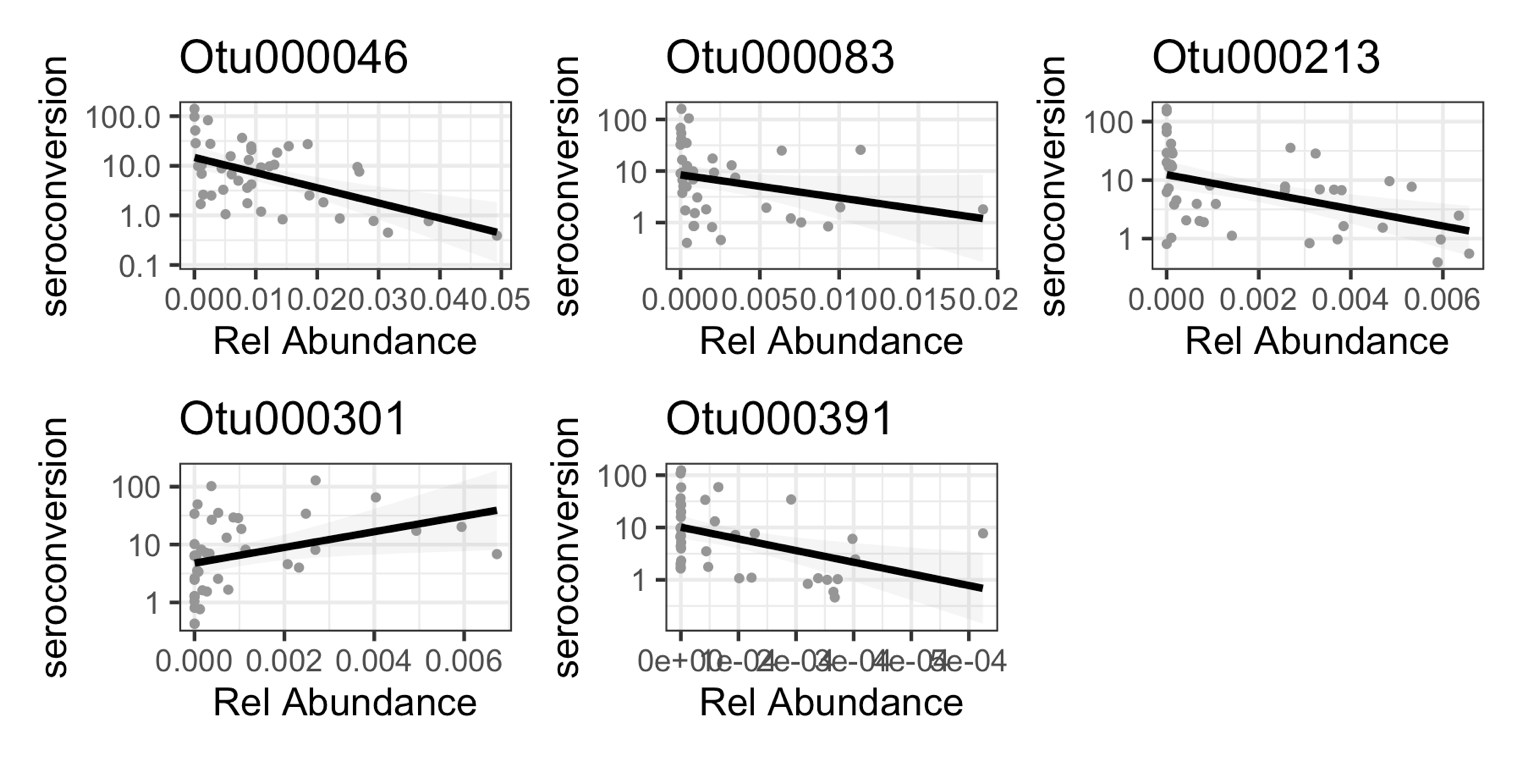


**Supplementary Figure 5:** Scatter plots of operational taxonomic units (OTUs) with a correlation coefficient > |0.15| that are significantly correlated with (A) H1N1 and (B) H3N2 seroconversion in participants naïve to previous vaccination. Rel = relative.


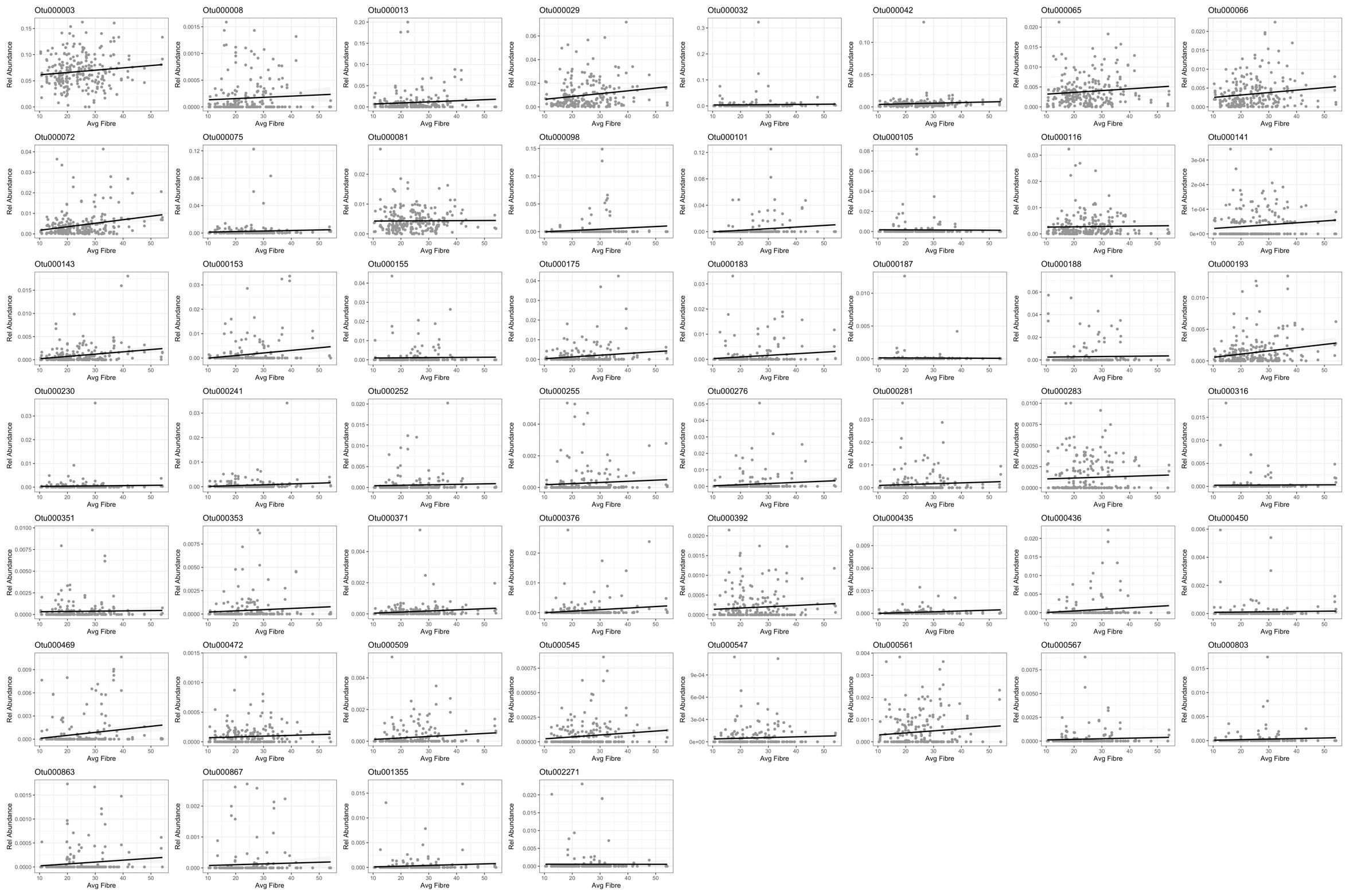


**Supplementary Figure 6:** Scatter plots of operational taxonomic units (OTUs) significantly correlated with average daily fibre consumption. Rel = relative. Avg = average.

**
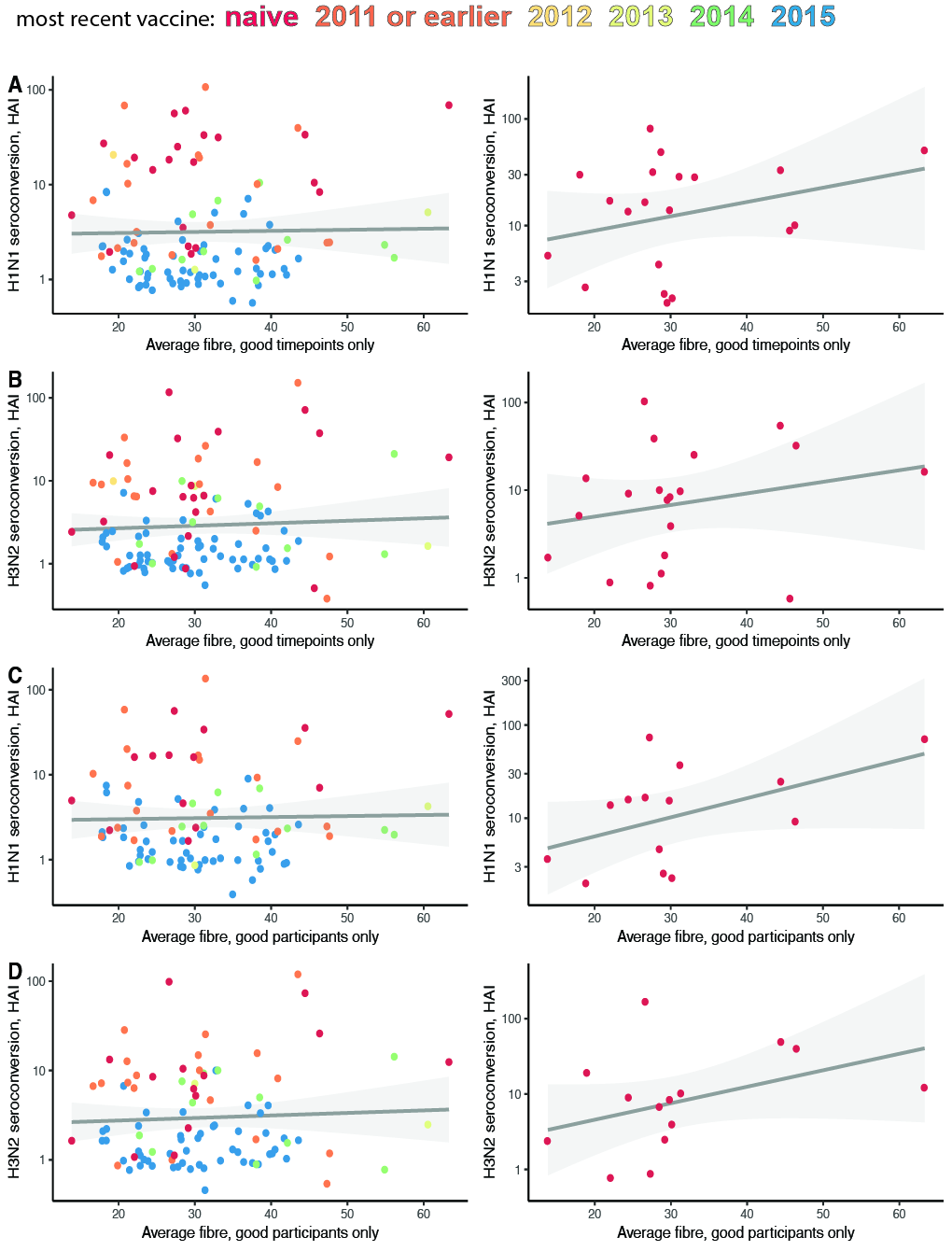
**

**Supplementary Figure 7. Fiber intake positively correlates with influenza vaccine responses.** Correlation analysis of average dietary fibre intake with Hemagglutinin (HAI) seroconversion. Colour represents the most recent year of self-reported seasonal influenza vaccination. On the left all participants are analyzed. On the right only participants naïve to previous influenza vaccination are analyzed. Grey shadow represents 95% confidence interval. To calculate average dietary fibre, we excluded: (A-B) individual days in which participants reported an energy intake less than 1.2 X the Schofield predicted base metabolic rate (“good timepoints only”). (C-D) any participant that did not report at least 3 days with an energy intake higher than 1.2 X the Schofield predicted base metabolic rate (“good participants only”).

**
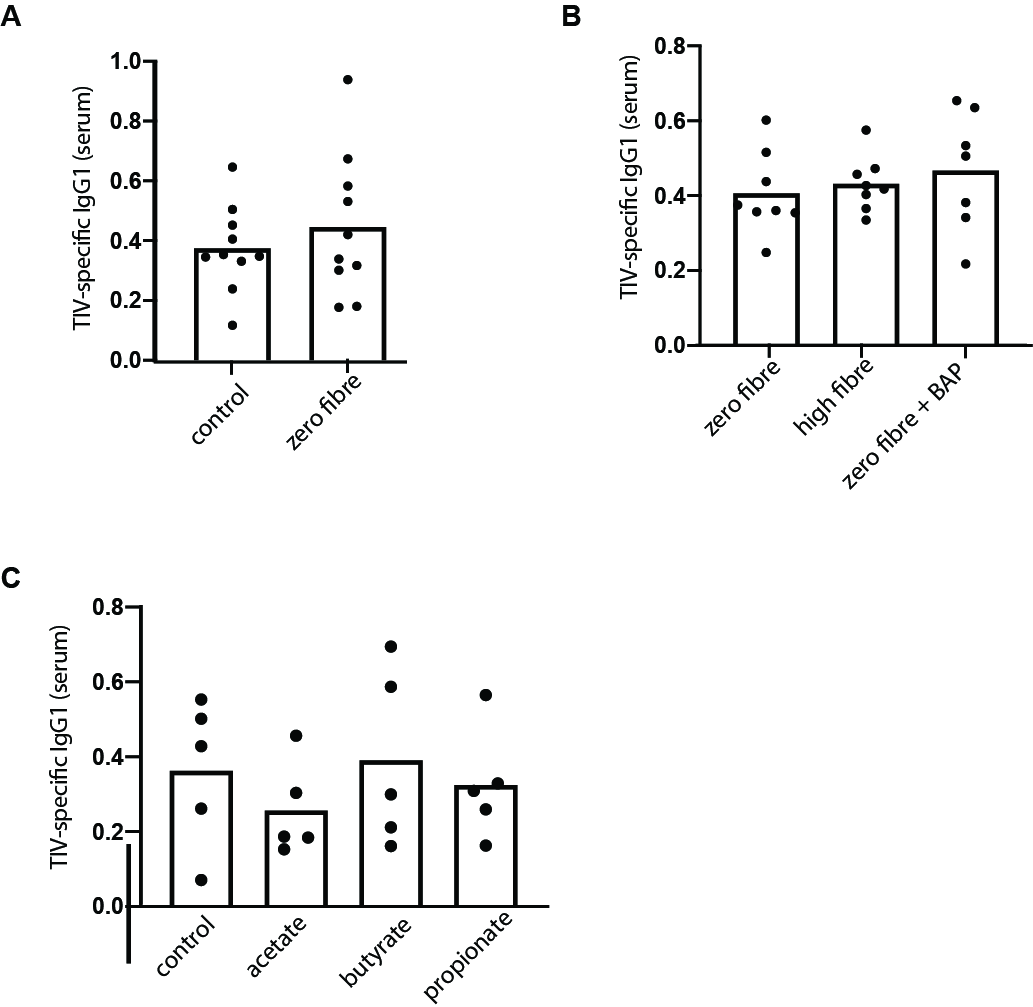
Supplementary Figure 8: Dietary fibre and fibre fermentation are not required for optimal IgG1 subclass response in a mouse model of influenza vaccination.** 28 days after receiving the trivalent influenza vaccine (TIV), serum levels of TIV-specific IgG1 were assessed from mice various fibre interventions. (A) TIV-specific IgG1 in the serum from mice fed a control or zero fibre diet. (B) TIV-specific IgG1 from mice on a zero-fibre diet, a high fibre diet, or a zero-fibre diet supplemented with a cocktail of short chain fatty acids containing butyrate, acetate, and propionate (C) TIV-specific IgG1 from mice on a control diet supplemented with acetate, butyrate, or propionate. Data from A and B are from two independent experiments, samples from replicate groups are combined for analysis ((A) n=5/treatment/ experiment (B) n=3-4/treatment/experiment). Data from C is representative from 2 independent experiments (n = 5/ treatment / experiment). Error bars in all panels show standard error of the mean. BAP = butyrate, acetate, and propionate.


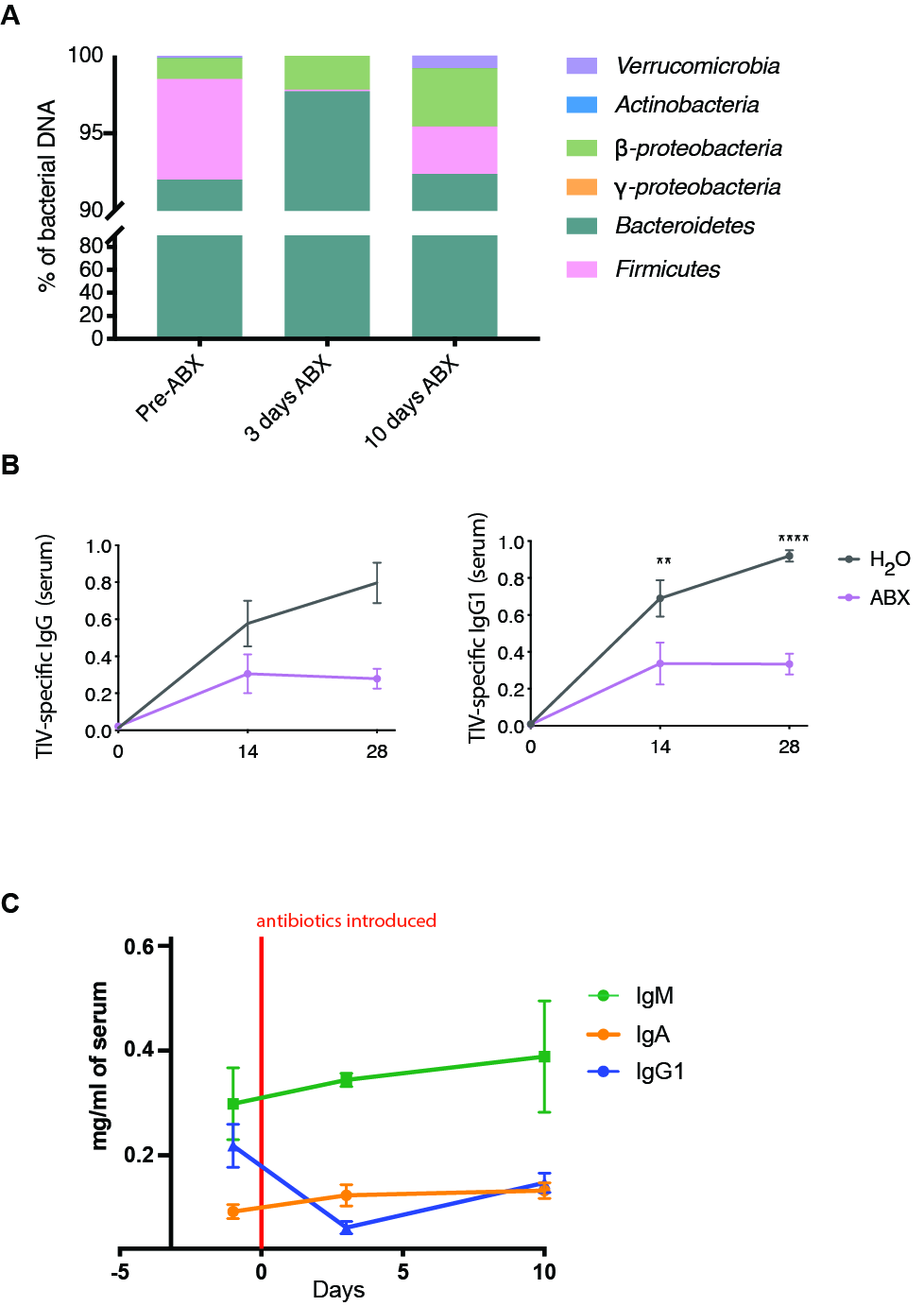


**Supplementary Figure 9: Effect of broad-spectrum antibiotics on humoral immunity and the microbiome in mice.** Data from mice on a control diet, with (abx) or without (control) the addition of an antibiotic cocktail to the drinking water. (A) Faecal-resident bacterial frequencies within a cage of mice was determined by qPCR using 16S rRNA gene-targeted group-specific primers for Bacteroidetes, Firmicutes, Proteobacteria, Actinobacteria, Verrucomicrobia, Candidatus Saccharibacteria, Deferribacteres and Tenericutes, and proportions were quantified against a universal 16S sequence. Proportions of bacteria are presented as the group mean. (B) Serum TIV-specific total IgG (left) and IgG1 (right) before vaccination, 14 days post vaccination, and 28 days post vaccination. (C) Total IgM, IgA, and IgG1 in the serum before the introduction of antibiotics, 3 days post antibiotics, and 10 days post antibiotics. Data shown is n=4-5 mice per group. Statistical analysis of antibody titres was performed using Two-Way ANOVA with Sidak’s posthoc test. Showing mean ± standard error of the mean. * P < 0.05, **P <0.01, *** P < 0.001, ****P<0.0001
